# Genesis of electron deficient Pt1(0) in PDMS-PEG aggregates

**DOI:** 10.1038/s41467-019-08804-y

**Published:** 2019-03-01

**Authors:** Kairui Liu, Guangjin Hou, Jingbo Mao, Zhanwei Xu, Peifang Yan, Huixiang Li, Xinwen Guo, Shi Bai, Z. Conrad Zhang

**Affiliations:** 1 State Key Laboratory of Catalysis, Dalian National Laboratory for Clean Energy, Dalian Institute of Chemical Physics, Chinese Academy of Sciences, 457 Zhongshan Road, 116023 Dalian, Liaoning China; 2 University of Chinese Academy of Sciences, 100049 Beijing, China; 3 State Key Laboratory of Fine Chemicals, PSU-DUT Joint Centre for Energy Research, School of Chemical Engineering, Dalian University of Technology, 116024 Dalian, China; 4 Department of Chemistry and Biochemistry, University of Delaware, Newark, DE 19716 USA; 5 State Key Laboratory of Applied Organic Chemistry, Lanzhou University, 730000 Lanzhou, Gansu China

## Abstract

While numerous single atoms stabilized by support surfaces have been reported, the synthesis of in-situ reduced discrete metal atoms weakly coordinated and stabilized in liquid media is a more challenging goal. We report the genesis of mononuclear electron deficient Pt_1_(0) by reducing H_2_PtCl_6_ in liquid polydimethylsiloxane-polyethylene glycol (PDMS-PEG) (Pt_1_@PDMS-PEG). UV–Vis, far-IR, and X-ray photoelectron spectroscopies evidence the reduction of H_2_PtCl_6_. CO infrared, and ^195^Pt and ^13^C NMR spectroscopies provide strong evidence of Pt_1_(0), existing as a pseudo-octahedral structure of (R^1^OR^2^)_2_Pt(0)Cl_2_H_2_ (R^1^ and R^2^ are H, C, or Si groups accordingly). The weakly coordinated (R^1^OR^2^)_2_Pt(0)Cl_2_H_2_ structure and electron deficient Pt_1_(0) have been validated by comparing experimental and DFT calculated ^195^Pt NMR spectra. The H^+^ in protic state and the Cl^−^ together resemble HCl as the weak coordination. Neutralization by a base causes the formation of Pt nanoparticles. The Pt_1_@PDMS-PEG shows ultrahigh activity in olefin hydrosilylation with excellent terminal adducts selectivity.

## Abstract (web summary)

Synthesis of discrete reduced metal atoms weakly coordinated and stabilized in liquid media remains challenging. Here, the authors report the genesis of mononuclear electron deficient Pt1(0) in polydimethylsiloxane-polyethylene glycol aggregates, and its ultrahigh activity in olefin hydrosilylation with excellent terminal adducts selectivity.

## Introduction

Supported single atoms have been prepared using various methods^1,2^. The reduced metal atoms are stabilized on solid surfaces through metal-support interaction^3,4^ or by strong ligand coordination^5,6^. The noble metal atoms are often capable of activating adsorbed reactants in catalysis^7,8^. Reduced single atoms tend to aggregate into nanoparticles in liquid media in the absence of strong ligand coordination^9^. Full and strong coordination is not desired for metal catalysts^10^. Therefore, synthesis of weakly coordinated and yet stable metal atoms in liquid media may pave a new path for facile preparation of highly dispersed metal catalysts by overcoming the long-standing challenge of metal aggregation. The readily removable ligands from the discrete metal atoms may also potentially enable atomically controllable synthesis of metallic materials by design.

Numerous surfactants with diverse functionalities have been used in the synthesis of metallic nanoparticles^11,12^ or metal ion-surfactant complexes^13,14^ in micro-emulsions system. The synthesis of discrete reduced metal atoms with weak coordination, which are sufficiently stable in storage at moderate temperature has not been reported. In this work, we chose polydimethylsiloxane-polyethylene glycol (PDMS-PEG) copolymer as an amphiphilic solvating agent. The hydrophilic and semicrystalline PEG blocks enable the dispersion of the hydrophobic PDMS blocks in aqueous solutions^15,16^. In our synthesis, ethanol is used as the reductant for Pt(IV)^17,18^, in the presence of optimized 10% (v/v) water to accelerate the rate of reduction^9^, while the diverse oxygen-containing functionality of PDMS-PEG offer weak coordination to the Pt atoms.

## Results & discussion

### Formation and spectroscopic characterizations of Pt_1_ (0) in ethanol reduction of ionic Pt precursors

**Fig. 1:**
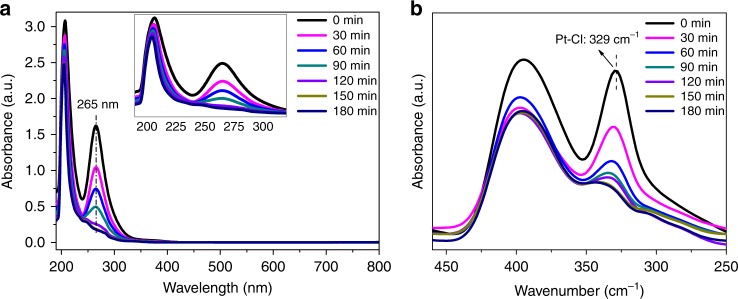
The reduction of H_2_PtCl_6_. **a** Ultraviolet (UV)–visible (Vis) spectra of H_2_PtCl_6_ during the ethanol reduction process. **b** Far infrared spectra in the process of H_2_PtCl_6_ reduction

Weakly coordinated mononuclear Pt_1_(0) (Pt_1_@PDMS-PEG) was prepared by ethanol reduction of H_2_PtCl_6_ in PDMS-PEG/ethanol−water (see Methods). The characteristic ultraviolet (UV)–visible (Vis) absorption peak of [PtCl_6_]^2−^ at 265 nm disappeared and that of [PtCl_4_]^2−^ anions at 220 nm was absent^17,18^ after the solution was refluxed for 3 h at 105 °C, indicating complete reduction of the Pt ions (Fig. 1a). While the decrease of the signal at 329 cm^−1^ in far infrared spectra^19,20^ (Fig. 1b) confirmed the loss of Coulombic Pt–Cl bonds, characterized by the chlorides of positively charged Pt ions, the retained Cl with a Cl/Pt atomic ratio of 2 in Pt_1_@PDMS-PEG was determined by chloride ion-chromatography analysis (Supplementary Table 1). Concomitant with the reduction of Pt(IV) (Supplementary Table 2), the color of the solution turned from bright yellow to colorless. Furthermore, the characteristic broad peak of Pt nanoparticles between 400 and 800 nm was not observed (Fig. 1a), indicating the absence of Pt_n_ clusters in the system^21^. When PDMS-PEG was replaced by either PDMS or PEG alone, a dark brown color indicative of Pt nanoparticle formation was observed under the same conditions^18^.

**Fig. 2:**
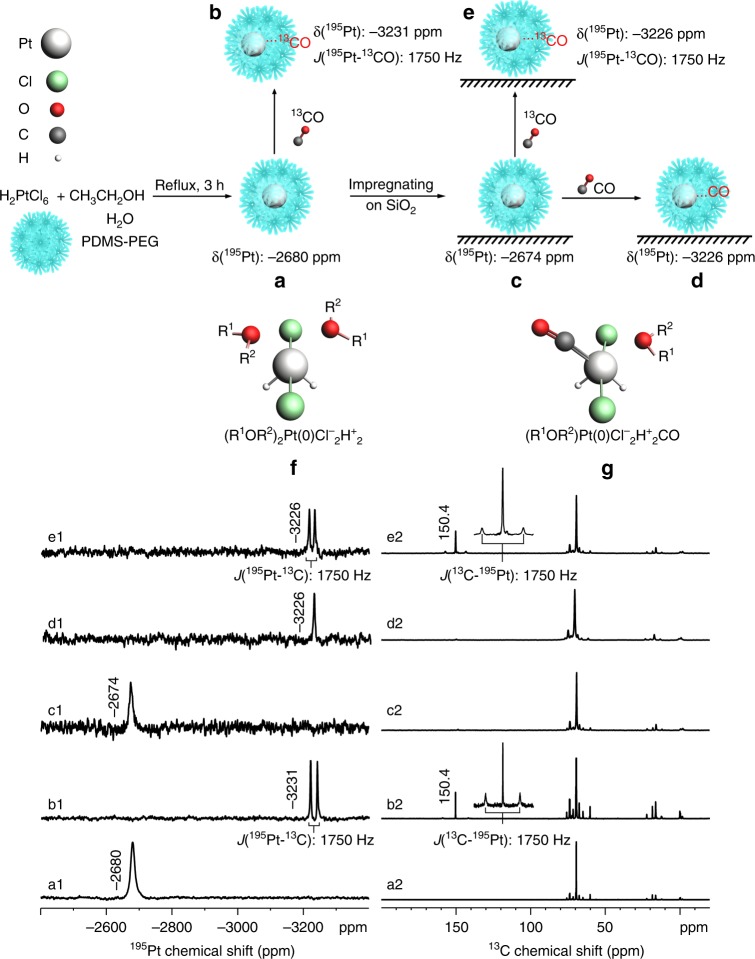
^195^Pt NMR (a1–e1) and corresponding ^13^C NMR (a2–e2) spectra of **a** Pt_1_@PDMS-PEG liquid; **b** ^13^CO treated Pt_1_@PDMS-PEG liquid; **c** 2 wt% Pt_1_@PDMS-PEG/SiO_2_; **d** CO treated 2 wt% Pt_1_@PDMS-PEG/SiO_2_; **e** ^13^CO treated 2 wt% Pt_1_@PDMS-PEG/SiO_2_. Note: 2 wt% refers to the Pt loading on SiO_2_; **f**, **g** are the selected density functional theory (DFT) models of *pseudo* octahedral structure of (R^1^OR^2^)_2_Pt(0)Cl^−^_2_H^+^_2_ (R^1^, R^2^ are H, C, or Si groups) and (R^1^OR^2^)Pt(0)Cl^−^_2_H^+^_2_CO in polydimethylsiloxane-polyethylene glycol (PDMS-PEG), respectively. Note: the sketch only serves as an illustration of possible local structures

The absence of ^195^Pt nuclear magnetic resonance (NMR) signals near 0 ppm^22,23^ for [PtCl_6_]^2−^ and −1617 ppm^24^ for [PtCl_4_]^2−^ confirms the full reduction of platinum. Meanwhile, a pronounced ^195^Pt NMR peak at −2680 ppm (Fig. 2a1) was observed and assigned to Pt_1_(0) in PDMS-PEG. This conclusion has been independently verified by the reduction of [PtCl_4_]^2−^ in PDMS-PEG monitored by ^195^Pt NMR (Supplementary Fig. 1). ^195^Pt NMR calculation of Pt(II) in PtCl_2_L_2_ or binuclear Pt_2_(μ-Cl)_2_Cl_2_L_2_ complexes with bridging Cl ligands (Supplementary Table 3) also supports the reduced state of Pt_1_ center^25^.

CO has been widely used to probe the dispersion of Pt particles on supports^4^. CO is known to coordinate to Pt by forming a linear Pt–CO bond or bridged bonds (Pt_2_CO and Pt_3_CO) on the surface of Pt clusters^4^. The identity of the discrete mononuclear state of Pt atoms was further verified by CO coordination using ^195^Pt and ^13^C NMR spectroscopy. ^13^CO treatment of Pt_1_@PDMS-PEG (Methods) led to an up-field shift of the ^195^Pt peak from −2680 to −3231 ppm as a doublet with a splitting of 1750 Hz, (Fig. 2b1), corresponding to one CO coordination to one Pt_1_(0) center. The ^13^C NMR spectrum (Fig. 2b2) of this sample also showed a new peak at 150.4 ppm with a ^195^Pt satellite doublet (^195^Pt-^**13**^**C**O) having the same splitting (1750 Hz). Since the ^13^C NMR chemical shift of solvated CO in various solvents ranges from 184.9 to 188.0 ppm^26^, the peak at 150.4 ppm can be assigned to the carbon of the newly formed Pt•••C = O species. In concert with the experimental observation, the density functional theory (DFT) calculation of ^13^C NMR chemical shift also shows a 24.1 ppm up-field shift from the gas phase CO to Pt•••C = O (Entries 8, 13 in Supplementary Table 4).

In both ^195^Pt and ^13^C NMR spectra, the splitting of 1750 Hz can be unambiguously assigned as the spin–spin coupling constant ^1^***J***(^195^Pt-^13^C). ^13^CO coordination to any form of multi-nuclear platinum cluster would produce more complex spin–spin coupling patterns than a simple doublet (Fig. 2b) of a linear Pt•••C = O structure. Thus, we conclude that the observed ^195^Pt NMR data unambiguously demonstrate the discrete mononuclear state of Pt atoms and Pt_1_•••C = O species, respectively.

When a concentrated Pt_1_@PDMS-PEG solution was purged with CO (Methods), a new peak at 341 cm^-1^ (vibration of Pt_1_-CO) in far infrared appeared^19,20^ (Supplementary Fig. 2). Altogether with the ^195^Pt NMR signal shift to −3231 ppm after the ^13^CO treatment, the results indicate that CO is a strong ligand. The ^195^Pt NMR linewidth of 265 Hz for Pt_1_•••C = O, almost fivefold reduction from that of solvated Pt_1_@PDMS-PEG, may be attributed to the enhanced mobility in the vicinity of Pt sites upon formation of the Pt•••C = O bond.

### Determination of coordination structure and electronic state of Pt_1_(0)

Extensive DFT-based structure screening using geometry optimization was followed by validation through comparing the calculated ^195^Pt chemical shifts of the selected structural models^27–29^ with the experimental shifts. Consistent with the fact that there are two chlorines per Pt_1_(0) in Pt_1_@PDMS-PEG based on the chloride ion chromatographic analysis, the DFT survey reveals a set of *pseudo* octahedral structures (R^1^OR^2^)_2_Pt(0)Cl^−^_2_H^+^_2_ (R^1^, R^2^ are H, C, or Si groups) (Entries 2–5, Supplementary Table 4) that not only stabilize Pt_1_(0), but also leads to the calculated ^195^Pt chemical shifts in reasonable agreement with the experimental value (−2680 ppm) for Pt_1_@PDMS-PEG.

In general, these structures contain two electron-donating oxygens (hydroxyl oxygens of water, ethanol as well as ester oxygens of siloxane and PEG segments) and an otherwise *cis*-divacant octahedral structure (Pt_1_H_2_Cl_2_) (Entry 1, Supplementary Table 4). The DFT calculation indicates the coordination of Pt_1_(0) by H, Cl, and O simultaneously prevents Pt_1_(0) from forming aggregates. We noted that various oxygen coordinated structures, excluding Cl and H and the structures in which H^+^ bound to Cl^−^ failed to produce a structure matching the observed ^195^Pt chemical shift (Supplementary Table 4). Moreover, the DFT calculation shows that the Bader charge of Pt atoms in the optimized structures (Entries 1–5, Supplementary Table 4) is around 0.6 eV, indicating the Pt_1_(0) atoms are electron deficient (Pt_1_^δ+^ (0 < *δ* < 1)), possibly associated with the partial electronegative charge of Cl^−^. The Bader charge of hydrogen is positive for all optimized (R^1^OR^2^)_2_PtCl_2_H_2_ structures (Entries 2–5, Supplementary Table 4), suggesting the H in (R^1^OR^2^)_2_PtCl_2_H_2_ is similar to labile proton rather than to hydride in nature. In the presence of abundant protons such as in residual H_2_O and OH groups of the solvent, the protic H in the (R^1^OR^2^)_2_PtCl_2_H_2_ could be readily hydrated, e.g. H_3_O^+^. The calculated ^195^Pt chemical shift (−2705 ppm) of (R^1^OR^2^)_2_Pt(0)Cl^−^_2_(H_3_O^+^)_2_ (Entry 6, Supplementary Table 4) is in an excellent agreement with the experimental shift (−2680 ppm), suggesting that (R^1^OR^2^)_2_Pt(0)Cl^−^_2_(H_3_O^+^)_2_ may exist as a hydrated form of (R^1^OR^2^)_2_PtCl^−^_2_H^+^_2_ in the system. This calculated result is a further validation of the existence of protic H, rather than a hydride. The absence of ^195^Pt-^1^H spin–spin couplings in the ^195^Pt spectra may be explained by the rapid chemical exchanges between the protic H in (R^1^OR^2^)_2_PtCl^-^_2_H^+^_2_ and abundant labile protons of water and hydroxyl protons in the system. Furthermore, the protic H in the (R^1^OR^2^)_2_Pt(0)Cl_2_H_2_ is consistent with a recent report that showed HCl acted as a weak ligand in the stabilization of Ir nanoparticles with the H in protic state^30^. The PtCl_2_^2−^ species quickly aggregated to form Pt nanoparticles in methanol−water solution^9^. In our system, the (R^1^OR^2^)_2_Pt(0)Cl^−^_2_H^+^_2_ is well stabilized by the PDMS-PEG matrix.

Besides the weak coordination of the dioxygen in the liquid, the (R^1^OR^2^)_2_Pt(0)Cl_2_H_2_ is acidic because of the protic H. We found that the Cl and H in PtCl_2_H_2_ are readily removable by neutralization with a base, accompanying with the release and subsequent destabilization of bare Pt_1_^δ+^ atoms even at ambient temperature. The sintering of Pt under such condition was evidenced by noting the color of the solution changing from colorless to dark brown, which apparently is not associated with Pt(II or IV) chlorides. The fact that no redox reaction occurred during the neutralization process confirmed the Pt_1_ in (R^1^OR^2^)_2_PtCl_2_H_2_ was in reduced state. The Pt coordination with H and Cl further verified by HBr exchange, which led an up-field ^195^Pt NMR chemical shift of (R^1^OR^2^)_2_PtBr^−^_2_H^+^_2_ to −3399 ppm (Supplementary Fig. 3), as was predicted by the calculated ^195^Pt NMR value (−3197 ppm, Entry 7, Supplementary Table 4).

To further verify the *pseudo* octahedral structures with a CO coordination as Pt•••C = O, we replaced one of oxygen-containing groups (R^1^OR^2^) with CO, followed by DFT geometry optimization and ^195^Pt NMR chemical shift calculation. The corresponding optimized structures (Entries 9–12, Supplementary Table 4) yielded the calculated ^195^Pt chemical shifts again in acceptable agreement with the experimental value (−3231 ppm) of Pt•••C = O. A model without proper oxygen coordination (Entry 8, Supplementary Table 4) failed to produce ^195^Pt chemical shift in conformity to the experimental value. Experimentally, the ^195^Pt chemical shift is up-field shifted by 551 ppm from Pt_1_@PDMS-PEG to the CO treated Pt_1_@PDMS-PEG. ^195^Pt chemical shift calculation shows that one CO coordination replacing one of the two R^1^OR^2^ coordination (Entries 9–12, Supplementary Table 4) led to an up-field shift ranging from 366 to 489 ppm compared to the corresponding dioxygen coordinated structures (Entries 2–5, Supplementary Table 4), in remarkable agreement with the experimental up-field shift value of 551 ppm. At the same time, replacing the first R^1^OR^2^ group with CO is energetically favored (Δ*E* = −0.18 eV). Further replacement of the remaining R^1^OR^2^ with a CO resulted in negligible energy difference (Δ*E* = −0.04 eV), consistent with fact that only one CO binds to Pt_1_^δ+^ atom. DFT calculation also shows that di-coordinated Pt(II) structures, *trans*-Pt(II)Cl_2_(CO)_2_ and *cis*-Pt(II)Cl_2_(CO)_2_, could not produce predicted chemical shifts in agreement with the experimentally observed value −3231 ppm (Supplementary Table 5). In addition, unlike (R^1^OR^2^)Pt(0)Cl_2_H_2_, the formation of dicarbonyl complex, Pt(II)Cl_2_(CO)_2_, from Pt(II)Cl_2_(R^1^OR^2^) is energetically favorable by the DFT calculations (Supplementary Table 5).

To demonstrate the versatility of preparing supported mononuclear electron-deficient Pt from the liquid Pt_1_(0) precursor, a Pt_1_@PDMS-PEG/SiO_2_ sample with Pt loading equivalent to 2.0 wt% with respect to SiO_2_ was prepared (see Methods). In comparison, a low Pt loading, e.g., 0.17 wt%, was essential for the direct preparation of Pt single atoms on oxides support^4^ to minimize Pt sintering due to its weak interaction with the support. Solid-state ^195^Pt magic angle spinning (MAS) NMR spectrum (Figure 2c1) of the Pt_1_@PDMS-PEG/SiO_2_ showed a peak at −2674 ppm, resembling that of Pt_1_@PDMS-PEG in terms of chemical shift. After CO treatment (see Methods), the ^195^Pt peak was shifted up-field to −3226 ppm (Figure 2d1). The same ^1^***J***(^195^Pt-^13^C) values were simultaneously observed in the ^195^Pt and ^13^C MAS NMR spectra (Figs. 2e1 and e2) upon ^13^CO treatment of the Pt_1_@PDMS-PEG/SiO_2_ sample, indicating that Pt_1_@PDMS-PEG was well preserved and stably dispersed on the silica surface even after “drying” under vacuum at 40 °C. Remarkably, the Pt_1_@PDMS-PEG and Pt_1_@PDMS-PEG/SiO_2_ samples remained stable for over six months in storage at ambient temperature as evidenced by almost identical ^195^Pt NMR spectra periodically measured.

**Fig. 3:**
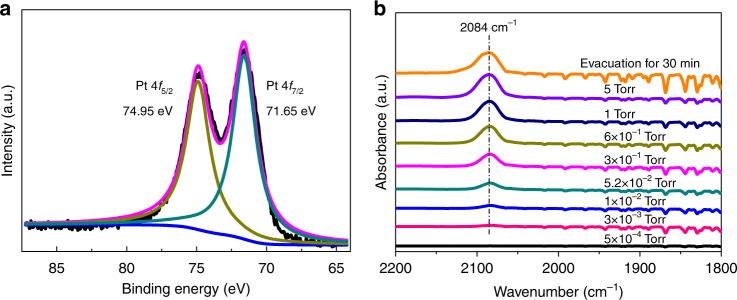
X-ray photoelectron spectra and CO adsorption of Pt_1_(0) atoms. **a** X-ray photoelectron spectra of 0.5 wt% Pt_1_@PDMS-PEG/SiO_2_, and **b** In situ diffuse reflectance infrared Fourier-transform (DRIFT) spectroscopy of CO adsorption on 1 wt% Pt_1_@PDMS-PEG/SiO_2_

To further verify the oxidation state of Pt after the reduction, a Pt_1_@PDMS-PEG liquid sample impregnated on pristine SiO_2_ was studied by X-ray photoelectron spectroscopy (XPS) (Fig. 3a). A peak at 71.65 eV (Pt 4*f*_7/2_), which is 0.45 eV higher than that of bulk Pt metal (71.20 eV)^31^ and 1.55 eV lower than that of Pt(II) (73.20 eV)^32^, confirmed the reduced state of Pt atoms in Pt_1_@PDMS-PEG/SiO_2_. Only one infrared (IR) absorption peak at 2084 cm^−1^ was observed for CO adsorbed on Pt_1_@PDMS-PEG/SiO_2_, but not on PDMS-PEG/SiO_2_ (Supplementary Fig. 4) by in situ diffuse reflectance infrared Fourier-transform (DRIFT) spectroscopy (Fig. 3b). Peak between 1800 and 1900 cm^−1^ for bridged CO was not observed, and the peak at 2084 cm^−1^ did not shift with increasing CO pressure, indicating the absence of nanoparticles in the system^4^. The DRIFT result is consistent with the NMR results of CO treated Pt_1_@PDMS-PEG (one-to-one coordination between CO and Pt_1_^δ+^ center), and the characteristic peaks at 2178 and 2138 cm^-1^ corresponding to the *cis*-Pt(II)Cl_2_(CO)_2_ were absent^33^.

The morphology of Pt_1_@PDMS-PEG solution was characterized by transmission electron microscopy (TEM). Fairly unilamellar vesicles with a narrow distribution (30–80 nm, Supplementary Fig. 5a) were formed^34^. The energy-dispersive X-ray spectroscopy analysis in a scanning transmission electron microscope (STEM) revealed the existence of Pt in the vesicles (Supplementary Fig. 5b and Supplementary Table 6). It should be noted that the Pt atoms tend to agglomerate under intense electron beam or X-ray radiation, making TEM, STEM, and synchrotron X-ray measurements infeasible in providing unambiguous evidence of discrete Pt atoms in our system^35^.

### Catalytic performance of electron deficient Pt_1_(0) in hydrosilylation reactions

An application of Pt_1_@PDMS-PEG as demonstrated in this work is its superb performance in hydrosilylation reaction^36,37^. Pt_1_@PDMS-PEG exhibited an ultrahigh activity with high selectivity (99%) to the terminal adduct in the benchmark hydrosilylation reaction of 1-octene and (Me_3_SiO)_2_MeSiH (Supplementary Table 7 and Supplementary Fig. 6). No 1-octene conversion was observed in a blank test using PDMS-PEG alone (Supplementary Table 7). The turnover frequency (TOF) of the Pt_1_@PDMS-PEG catalyst is 2 to 4 orders of magnitude higher than that of Karstedt^38^, NHC-Pt complexs^37,39^, and silylene-Pt complexs^38,40^ (Supplementary Table 7 and Supplementary Fig. 7). The turnover number (TON) exceeded 5.0 × 10^8^ for the hydrosilytion reaction of 1-octene using (Me_3_SiO)_2_MeSiH as the silylation reagent. Induction period of the hydrosilylation by Pt_1_@PDMS-PEG was negligible. The reaction solution remained colorless and transparent throughout hydrosilylation reaction.

The ultrahigh activity of Pt_1_@PDMS-PEG catalyst may be attributed to electron deficiency of the Pt_1_^δ+^ center^41^ and the reversible weakly electron-donating R^1^OR^2^ ligands as compared to the 1,3-divinyl−1,1,3,3-tetramethyldisiloxane (dvtms) or other olefin ligands. The Bader charge (~0.6 eV) on the Pt_1_^δ+^ center in (R^1^OR^2^)_2_PtCl_2_H_2_ (Entries 2–5, Supplementary Table 4) is much higher than the charge (0.13 eV) for Pt in the tri-olefin coordinated complex (Entry 9, Supplementary Table 8), representing Karstedt Pt catalyst. ^195^Pt chemical shift of the Pt_1_@PDMS-PEG is about 3450 ppm down-field shifted to that of the Karstedt Pt catalyst^42^, also in conformity with the high electron deficiency of the Pt_1_@PDMS-PEG catalyst. The dvtms coordinated Pt catalysts are typically characterized by a relative long induction period partly due to the slow substitution of dvtms by reactants^39^.

When 1-octene was added to Pt_1_@PDMS-PEG, the ^195^Pt NMR peak showed an insignificant up-field shifted by only 26 ppm to −2706 ppm (Supplementary Fig. 8). DFT calculation shows that only mono-olefin complex (olefin)_1_(R^1^OR^2^)_1_PtCl^−^_2_H^+^_2_ produces a ^195^Pt NMR chemical shift that reasonably agrees with the experimentally observed value (Entry 2, Supplementary Table 8). All structures with multiple olefin coordination fail to predict the experimental ^195^Pt NMR chemical shift. The dvtms stabilized Pt catalysts were reported to form colloidal platinum species, accompanied by the change of the solution color to yellow during the hydrosilylation catalysis process^43,44^, making the nuclearity of the active Pt catalyst debatable. In this work, in addition to the invariant color throughout the hydrosilylation reaction, the ^195^Pt NMR of the reaction solution after the catalytic reaction shows that the catalyst remained in mononuclear Pt_1_(0) state (Supplementary Fig. 9, *n*_1-octene_:*n*_silane_ = 5:3), suggesting that the highly durable mononuclear Pt_1_(0) center is the active site in Pt_1_@PDMS-PEG for the reaction. Moreover, through three recycled uses, Pt_1_@PDMS-PEG maintained high activity without noticeable deactivation in 1-octene hydrosilylation with (Me_3_SiO)_2_MeSiH (Supplementary Fig. 10). The color of the reused Pt_1_@PDMS-PEG solution remained clear throughout the reuses.

### Conclusion

We prepare discrete Pt_1_@PDMS-PEG through ethanol reduction of H_2_PtCl_6_. UV–Vis, Fourier-transform infrared spectroscopy (FTIR), ^195^Pt, and ^13^C NMR and XPS spectroscopies provide strong evidence for the formation of Pt_1_(0). Based on the extensive DFT structure screening and by comparing the calculated and experimental ^195^Pt NMR chemical shifts, a pseudo octahedral Pt_1_(0) structure, (R^1^OR^2^)_2_Pt(0)Cl^−^_2_H^+^_2_ with weakly coordinated Pt_1_(0) is proposed. Pt_1_@PDMS-PEG shows high thermal stability in solution and on support in a moderate temperature range. As a catalyst, it exhibits ultrahigh activity and durability for hydrosilylation with superb selectivity to terminal adduct. The electron-deficient Pt_1_^δ+^ center together with weak oxygen coordination may contribute to the high performance in hydrosilylation. Pt_1_@PDMS-PEG could be a potential new generation of hydrosilylation catalyst.

## Methods

### Chemicals

1-Octene (98%), silica, and PEG-200 were purchased from Aladdin Industrial Corporation. PDMS-2000 were purchased from Alfa Aesar (China) Chemicals Co., Ltd. *N*, *N*-dimethylaniline (99%) and 1,1,1,3,5,5,5-heptamethyltrisiloxane (98%) were purchased from Tokyo Chemical Industrial Co., LTD. Hydrochloric acid, chloroplatinic acid, ethanol, and ammonium hydroxide were purchased from Sinopharm Chemical Reagent Co., Ltd. PDMS-PEG was purchased from Guangzhou Tinci Materials Technology Co., Ltd. The molecular weight of each block is 30,000 for PDMS and 545 for PEG-12. Ultrapure water purified by Milli-Q Advantage A10 was used.

### Sample preparation

Preparation of Pt_1_@PDMS-PEG stock solution by reduction of H_2_PtCl_6_ with ethanol: PDMS-PEG (0.6465 g), ethanol (135 ml), and ultrapure water (10.2 ml) were stirred in a round-bottom flask at 450 rpm at room temperature (RT). After 10 min, 4.8 ml H_2_PtCl_6_ solution (1.84 × 10^−2 ^mol l^−1^) was added, and the mixture was stirred for 10 more minutes. The solution was refluxed at 105 °C for 3 h and then cooled to RT. (For NMR characterization, ethanol and water were evaporated at 40 °C under reduced pressure).

^13^CO treatment of Pt_1_@PDMS-PEG: Ethanol and water were removed under vacuum from the Pt_1_@PDMS-PEG stock solution at 40 °C. The Pt_1_@PDMS-PEG sample was purged with He for 30 min (100 ml min^−1^), followed by admission of 5% ^13^CO/He (15 ml min^−1^) for another 30 min. The sample was flushed with He for 1 h to remove free ^13^CO and collected in a glovebox under N_2_.

CO treatment of Pt_1_@PDMS-PEG for Far-IR (FIR) characterization: Ethanol and water were removed under vacuum from the Pt_1_@PDMS-PEG stock solution at 40 °C. The Pt_1_@PDMS-PEG sample was purged with He for 30 min (100 ml min^−1^), followed by admission of 5% CO/He (15 ml min^−1^) for another 30 min. The sample was flushed with He for 1 h to remove free CO and collected in a glovebox under N_2_ before the FIR characterization.

Preparation of Pt_1_@PDMS-PEG/SiO_2_: A calculated amount of silica based on the desired final Pt loading was added to 150 ml Pt_1_@PDMS-PEG stock solution and stirred for 1 h. Ethanol and water were removed at 40 °C under vacuum. The sample was further vacuum dried for 12 h at 40 °C. For DRIFT experiment and catalytic reactions, the pH value of the Pt_1_@PDMS-PEG stock solution was adjusted to 3.2 by NH_3_•H_2_O solution before the evacuation.

^13^CO treatment of Pt_1_@PDMS-PEG/SiO_2_: In a quartz tube 200 mg Pt_1_@PDMS-PEG/SiO_2_ was purged with He for 30 min (100 ml min^−1^) and 5% ^13^CO/He (15 ml min^−1^) for another 30 min. The sample was flushed with He for 1 h to remove free gas phase ^13^CO and collected in a glovebox under N_2_.

1-Octene disposed Pt_1_@PDMS-PEG: Concentrated 150 ml Pt_1_@PDMS-PEG solution to 20 ml. Added 0.0990 g 1-octene (ten times equivalent to Pt_1_) and 6 ml CHCl_3_ into the 20 ml concentrated solution (the use of CHCl_3_ is to enhance the 1-octene dissolution in the system). Stirred for 24 h at 40 °C and followed by further concentrating the solution to about 3 ml. The sample was collected in a glovebox under N_2_ prior to the ^195^Pt NMR measurement.

### Ultraviolet (UV)–visible (Vis)

UV–Vis spectra was collected on a Shimadzu UV-2600 spectrophotometer.

### X-ray photoelectron spectra (XPS)

The surface composition and the binding energy (B.E.) of the catalyst were determined by XPS on an ESCALAB250 X-ray photoelectron spectrometer with contaminated C as the internal standard (C1s = 284.6 eV).

### NMR spectroscopy

All solid-state NMR measurements were carried out on a 11.7 T wide-bore Bruker Avance III solid-state NMR spectrometer, operating at a Larmor frequency of 500.13 MHz for ^1^H, 125.77 MHz for ^13^C, and 107.21 MHz for ^195^Pt. A 4.0 mm Bruker multinuclear HX double resonance magic angle spinning (MAS) probe was used. The experiments were acquired at 25 °C and an MAS frequency of 10 kHz, controlled to within +/−3 Hz using the Bruker MAS controller. The typical 90° pulse length was 2.5 μs (^1^H), 3.6 μs (^13^C), and 4.2 μs (^195^Pt). Seventy-six thousand two-hundred scans were collected for ^195^Pt MAS NMR spectra with a recycle delay of 1 s and 30° pulse excitation (1.4 us). Eight-thousand one-hundred ninety-two scans were collected for ^13^C MAS NMR spectra with a recycle delay of 3 s, where ^1^H SPINAL-64 heteronuclear decoupling with field strength of 90 kHz was applied during the acquisition period. Solution NMR spectra were obtained on Bruker Avance III 600 MHz and 400 MHz using the 5 mm multinuclear probe. Single pulse and proton decoupling were used for ^195^Pt and ^13^C measurements with 3-s relaxation delay, respectively. The solution and solid-state ^195^Pt NMR spectra were referenced to a platinum peak of a 1.2 M Na_2_PtCl_6_ D_2_O solution. The chemical shifts of ^1^H and ^29^Si NMR spectra were referenced externally to TMS.

### Fourier-transform infrared spectroscopy (FTIR)

A DiffusIR diffuse reflectance accessory (Pike Technologies) was used for the FTIR measurements. A leveled attenuated total reflectance (ATR) accessory (Pike Technologies) with a 3 mm diameter diamond plate was used for Middle IR (MIR) and FIR measurements. The FIR instrument (Thermo Scientific Nicolet iS50) is equipped with mercury cadmium telluride (MCT) detector and DTGS/polyethylene detector. The DRIFT measurement has a resolution of 4 cm^−1^ and 64 scans were collected, while for FIR measurements, 128 scans were collected with a resolution of 16 cm^−1^. For the DRIFT measurements, the sample was evacuated at 25 °C for 1 h before absorption of high purity probing CO gas. For the FIR measurements, a steady flow of nitrogen was maintained and a high-temperature vacuum-grease-sealed glass lid was used above the sample to prevent air and moisture. Before the FIR experiment, ethanol and water in all the samples were removed by evacuation.

### Transmission electron microscopy (TEM) and scanning transmission electron microscope (STEM)

STEM analyses were performed with a JEOL JEM-2100F microscope operated at 200 kV. TEM analyses were performed with a JEOL JEM-2000EX microscope operated at 120 kV. A few droplets of the stock solution were put on a microgrid carbon polymer supported on a copper grid and allowed to dry out at room temperature for TEM and STEM observations. The samples were evacuated under ultrahigh vacuum for 20 min in the microscope before observations. For TEM characterization, phosphotungstic acid was used as the staining reagent.

### Chloride ion analysis by ion-chromatography

A THERMOICS-5000 ion-chromatography with the Electrochemical Detector (ED) and the AS-11 and AS-22 columns ware used for the detection of Cl^−^ in Pt_1_@PDMS-PEG. Before detection, a 5 ml of Pt_1_@PDMS-PEG was diluted to 10 ml with water to reach an appropriate concentration for ion chromatographic analysis.

### Computation of NMR shifts

The Amsterdam Density Functional (ADF) approach^45^, by which the relativistic effects for calculating of chemical shielding of heavy atom such as platinum can be implemented, was used. The relativistic effects were treated in the ADF as all-electron zeroth-order regular approximation (ZORA). For the chemical shielding, the spin-orbital (SO) two-component was used while for the geometry optimization the scalar approximation neglecting SO coupling was utilized. More specifically, as suggested by Autschbach et al.^27–29^, the revised Perdew-Burke-Ernzerhof (revPEB) functional was used for all calculations, and the Slate-type of basis sets (TP2Z) were used for ligand atoms and the quadruple-quadruply polarized (QZ4P) ZORA basis was set for Pt, respectively. The calculated chemical shift of the optimized structures was referenced to the calculated isotropic shielding constant (*σ*^ref^ = 2005 ppm) of the reference compound [PtCl_6_]^2−^ by the definition of *δ* = (*σ*^ref^ − *σ*)/(1 − *σ*^ref^) ≈ (*σ*^ref^ − *σ*) with an assumption that (1 − *σ*^ref^) ≈ 1. The geometry of [PtCl_6_]^2^ was optimized (*d*_Pt-Cl_ = 2.392 Å) using the same method described above prior to the calculation of the shielding constant (*σ*^ref^). Similar ADF method with scalar approximation neglecting SO coupling was used to calcuate ^13^C chemcial shielding of gas phase CO and Pt-coordinated CO in various structures.

### General procedure for hydrosilylation

We followed Iwamoto’s procedure^38^ to evaluate the Pt_1_@PDMS-PEG as the catalyst for the hydrosilylation reaction of 1-octene with 1,1,1,3,5,5,5-heptamethyltrisiloxane (Supplementary Table 7). In all, 0.034 ml Pt_1_@PDMS-PEG stock solution (Pt concentration of 5.88 × 10^−4 ^mol l^−1^) was added into the reactor. Ethanol and water were evacuated at RT. All reactions were carried out in glovebox. 1-Octene (4 mmol) was added and mixed with the Pt_1_@PDMS-PEG [corresponding to *n*(1-octene):*n*(Pt_1_) = 200,000:1] for 3 min at 25 °C before reaction. 1,1,1,3,5,5,5-heptamethyltrisiloxane (4.4 mmol) was then added, and the reaction was carried out at 50 °C. After reaction, 2 mmol *N*, *N*-dimethylaniline (0.2424 g) was added as an internal standard. Products and reactants were characterized by ^1^H NMR (400 MHz, CDCl_3_, and Supplementary Fig. 6). Blank test was run as follows: hydrosilylation reaction was carried out with PDMS-PEG/ethanol-water as the catalyst (without Pt) following the same procedure as for the hydrosilylation of 1-octene catalyzed by Pt_1_@PDMS-PEG, No conversion of 1-octene was observed (Supplementary Table 7).

The reported conversion measurement was repeated at least three times under the same condition, and the uncertainty was typically below 5%. The average value of multiple measurements is presented in the paper.

The turnover frequency (TOF), defined as the number of moles of terminal product (**T**) yield per mole of Pt_1_(0) within a unit time, is calculated in unit of h^−1^ according to

$$\begin{array}{*{20}{l}} {{\mathrm{TOF}}} \hfill & = \hfill & {\frac{{n_{\mathrm{T}}}}{{n_{{\mathrm{Pt}}}}} \times \frac{1}{t}} \hfill \\ {} \hfill & = \hfill & {\frac{{n_{{\mathrm{olefin}},0} \times {\mathrm{yield}}_{\mathrm{T}}}}{{n_{{\mathrm{Pt}}}}} \times \frac{1}{t}} \hfill \end{array}$$

Since the conversion reached nearly completion in a minute, the initial TOF was calculated by taking *t* as 1 min for the catalytic reaction under consideration.

*n*_olefin,0_ is the initial number of moles of 1-octene used in the reaction. Note that *n*_1−octene,0_ is 4 mmol.

*n*_T_ is the number of moles of product, T (terminal adduct).

*n*_Pt_ is the number of moles of Pt added in the reaction.

*t* is the reaction time. The conversion value was calculated at *t* = 1 min.

$${\mathrm{yield}}_{\mathrm{T}} = {\mathrm{conversion}} \times {\mathrm{selectivity}}$$

### Turnover numbers (TONs)

TON of Pt_1_@PDMS-PEG in the hydrosilylation reaction between 1-octene and 1,1,1,3,5,5,5-heptamethyltrisiloxane: 0.0068 ml Pt_1_@PDMS-PEG stock solution (diluted 10 times, 5.88 × 10^−5^ mol l^−1^, 4 × 10^−10^ mol) was added into the round-bottom flask, followed by evacuation of ethanol-water. All reaction was carried out in glovebox. 1-Octene (40 mmol) was added and mixed with the Pt_1_@PDMS-PEG [*n*(1-octene):*n*(Pt_1_) = 1 × 10^8^: 1] for 3 min at 25 °C before reaction. 1,1,1,3,5,5,5-heptamethyltrisiloxane (48 mmol) was then added, and the mixture was refluxed at 70 °C. After the completion of the reaction (verified by ^1^H NMR), add more 1-octene and 1,1,1,3,5,5,5-heptamethyltrisiloxane (1:1.2) into the reactor and continue the reaction.

### Catalyst recycle

After the hydrosilylation reaction between 1-octene and (Me_3_SiO)_2_MeSiH (1-octene: 0.4488 g, (Me_3_SiO)_2_MeSiH: 0.89 g) finished, the solution was centrifuged. In all, 0.6694 g reaction mixture was taken out from the upper solution, and analyzed by ^1^H NMR. The remaining solution (0.6694 g) was used for the subsequent hydrosilylation reaction. For the reuse test, 0.4488 g 1-octene and 0.89 g (Me_3_SiO)_2_MeSiH was added. After reaction, solution was centrifuged. In all, 1.3388 g (equal to the amount of added reactants) reaction mixture was taken out from the upper solution, and analyzed by ^1^H NMR, while the remaining solution (0.6694 g) was used for the subsequent hydrosilylation reaction. For the following reuses, the added amounts of 1-octene and (Me_3_SiO)_2_MeSiH were always 0.4488 and 0.89 g, respectively. After each reaction, the amount of reaction mixture taken out for ^1^H NMR from the upper solution after centrifugation was always 1.3388 g (equal to the amount of added reactants), and the remaining solution was kept at 0.6694 g for the subsequent hydrosilylation reaction.

## Supplementary information

Supplementary Information

Peer Review File

## Data Availability

The data that support the findings in this study are in the published article and/or its Supplementary Information files. The whole datasets are available from the corresponding author on reasonable request. The source data are provided as a Source Data file.
