# Supplementary material for: Genesis of electron deficient Pt1(0) in PDMS-PEG aggregates
Source: Nat Commun. 2019 Mar 1;10:996. doi: 10.1038/s41467-019-08804-y (PMC6397160; doi:10.1038/s41467-019-08804-y)
Supplement: Supplementary file 1 — Supplementary Information [file 41467_2019_8804_MOESM1_ESM.pdf]

Genesis of Electron Deficient $Pt_1(0)$ in PDMS-PEG Aggregates

Liu et al.

Table of Content

1. Cl/Pt molar ratio in Pt_1@PDMS-PEG 1
2. Reduction equation of H_2PtCl_6....1
3. DFT calculation, ^{195}Pt NMR of $PtCl_2L_2$ and $Pt_2(\mu\text{-}Cl)_2Cl_2L_2$....2
4. DFT calculation, ^{195}Pt NMR and Bader charge of $(R^1OR^2)_2Pt(0)Cl^-{}_2H^+{}_2$....5
5. DFT calculation, ^{195}Pt NMR of $PtCl_2(CO)_2$ and cis-$PtBr_2(CO)_2$....9
6. EDX analysis of Pt_1@PDMS-PEG....11
7. Catalytic performance....12
8. DFT calculation and ^{195}Pt NMR of $(olefin)_1(R^1OR^2)_1Pt(0)Cl^-{}_2H^+{}_2$ 13
9. ^{195}Pt NMR of K_2PtCl_4 reduction....15
10. Far infrared spectrum of Pt_1-CO@PDMS-PEG....16
11. ^{195}Pt NMR of HBr exchanged Pt_1@PDMS-PEG....17
12. DRIFT spectra of CO absorption on PDMS-PEG/SiO_2....18
13. TEM and STEM of Pt_1@PDMS-PEG....19
14. ^1H NMR spectrum of reaction mixture.... 20
15. Catalytic performance comparison....23
16. ^{195}Pt NMR spectrum of $(olefin)(R^1OR^2)Pt(0)Cl^-{}_2H^+{}_2$....24
17. ^{195}Pt NMR spectrum of the catalyst after hydrosilylation 25
18. Reusability test of catalyst....26

Supplementary Tables

Supplementary Table 1. Cl/Pt molar ratio in Pt_1@PDMS-PEG

Initial Cl^- concentration in H_2PtCl_6 (mg L^{-1}) (M_{Cl})[a]	62.60
Free Cl^- concentration after reduction (mg L^{-1})[b]	41.52
Number of Cl^- per $Pt_1(0)$ (n_{Cl})[c]	2.0

[a] Calculation method: in the preparation of Pt_1@PDMS-PEG, 4.8 ml of 0.0184 mol L^{-1} H_2PtCl_6 was added into 145.2ml PDMS-PEG/ethanol-water solution. Before ion chromatographic measurement, 5 ml Pt_1@PDMS-PEG was diluted to 10 ml with water. Thus:

Initial Cl^- concentration in H_2PtCl_6: $M_{Cl} = \frac{4.8 \times 0.0184}{4.8+145.2} \times 6 \times 35.45 \div 2 = 62.60\, mg\ \mathrm{L}^{-1}$,

where 35.45 g mol^{-1} is the chlorine atomic weight.

[b] Only free Cl^- can be detected by chloride ionchromatography.

[c] Number of Cl^- coordinated to Pt: $n_{Cl} = 6 \times (1 - 41.52/62.60) = 2.0$

Supplementary Table 2. Equation of the reduction of H_2PtCl_6

		$E^{\ominus}/V$
(1)	$[PtCl_6]^{2-}$ + CH_3CH_2OH = $[PtCl_4]^{2-}$ + CH_3CHO + $2H^+$ + $2Cl^-$	0.68
(2)	$[PtCl_4]^{2-}$ + CH_3CH_2OH = Pt_1^0 + CH_3CHO + $2H^+$ + $4Cl^-$	0.755

Supplementary Table 3. DFT structure optimization[a] and calculation of NMR chemical shift of $PtCl_2L_2$ and $Pt_2(\mu\text{-}Cl)_2Cl_2L_2$ (L= R^1OR^2).

**Entry**	**Structures**	**^{195}Pt Chemical shift[b] and key structure parameters**	**Entry**	**Structures**	**^{195}Pt Chemical shift[b] and key structure parameters**
**1**	Pt(II)Cl_2 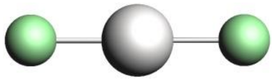	δ(^{195}Pt): 60218 ppm<br>Pt–Cl: 2.16 Å	**6**	*trans*-$PtCl_2$-2CH_3OCH_3 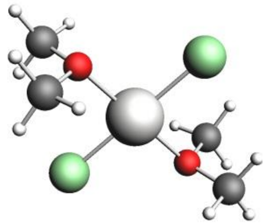	δ(^{195}Pt): -1442 ppm<br>Pt–Cl: 2.32 Å<br>Pt–O: 2.06 Å
**2**	*trans*-$PtCl_2$-2H_2O 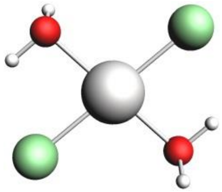	δ(^{195}Pt): -1427 ppm<br>Pt–Cl: 2.31 Å<br>Pt–O: 2.06 Å	**7**	*cis*-$PtCl_2$-2CH_3OCH_3 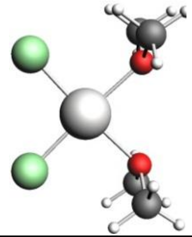	δ(^{195}Pt): -1439 ppm<br>Pt–Cl: 2.26 Å<br>Pt–O: 2.15 Å
**3**	*cis*-$PtCl_2$-2H_2O 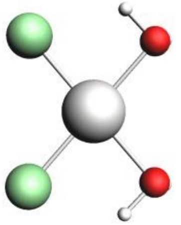	δ(^{195}Pt): -1387 ppm<br>Pt–Cl: 2.26 Å<br>Pt–O: 2.14 Å	**8**	$PtCl_2$-$SiO_4(CH_3)_4$ 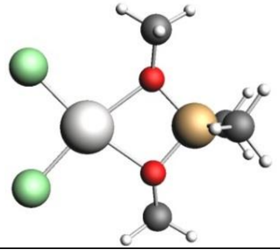	δ(^{195}Pt): -1076 ppm<br>Pt–Cl: 2.25 Å<br>Pt–O: 2.17 Å

**4**	*trans*-$PtCl_2$-$2C_2H_5OH$ 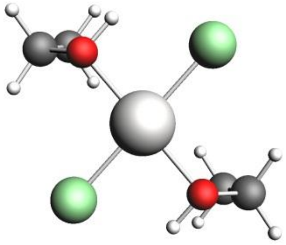	δ(^{195}Pt): - 1569 ppm<br><br>Pt–Cl: 2.31 Å<br>Pt–O: 2.05 Å	**9**[c]	$Pt_2(\mu\text{-}Cl)_2Cl_2(H_2O)_2$ 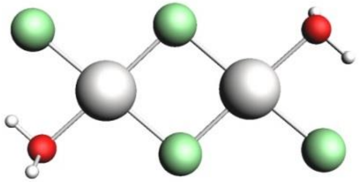	δ(^{195}Pt): -1255 ppm<br><br>Pt–Cl: 2.28/2.35 Å<br>Pt–O: 2.11 Å
**5**	*cis*-$PtCl_2$-$2C_2H_5OH$ 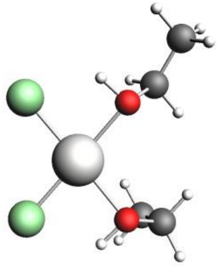	δ(^{195}Pt): -1572 ppm<br><br>Pt–Cl: 2.27 Å<br>Pt–O: 2.13 Å	**10**[c]	$Pt_2(\mu\text{-}Cl)_2Cl_2(C_2H_5OH)_2$ 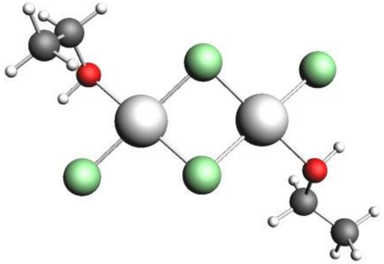	δ(^{195}Pt): -1363 ppm<br><br>Pt–Cl: 2.29/2.35 Å<br>Pt–O: 2.09 Å
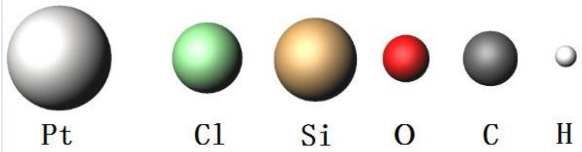					

[a] The chemical structure of PDMS-PEG is given below:

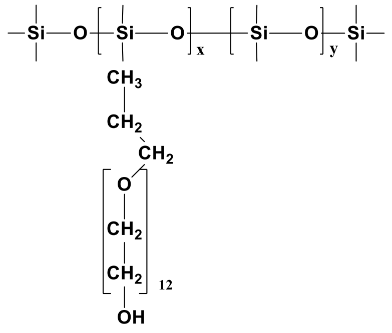

In structure optimization, the coordinations of these structures to the oxygens of water and ethanol (Entries 2-5) were considered. The MeOMe and $Si(Me)_2(OMe)_2$ were used to represent the segment structure of–O–C–C–O in PEG (Entries 6, 7) and -O-Si-O- in PDMS (Entry 8), respectively.

[b] The calculated $\delta(^{195}Pt)$ is referenced to $[PtCl_6]^{2-}$ by the definition of $\delta=(\sigma^{ref}-\sigma)/(1-\sigma^{ref}) \approx (\sigma^{ref}-\sigma)$ with an assumption that $(1-\sigma^{ref}) \approx 1$. The calculated isotropic nuclear shielding constant $\sigma(^{195}Pt)$ of this reference is 2005 ppm using the same computational method described in "DFT geometry optimization and ^{195}Pt and ^{13}C chemical shift calculation" of **Methods.**

[c] For the $Pt_2(\mu\text{-}Cl)_2Cl_2L_2$, only ethanol and water were considered.

The calculated ^{195}Pt chemical shift for $Pt(II)Cl_2(R^1OR^2)_2$ (Entries 2-8, **Supplementary Table 3**) are in a range of -1076 to -1572 ppm, in reasonable agreement with the experimental ^{195}Pt chemical shift of Pt(II) ($[PtCl_4]^{2-}$) in the range of -1485 and -1617 ppm[24], that is distinctively different from the experiment value (-2680 ppm) of Pt_1@PDMD-PEG. The calculated ^{195}Pt chemical shifts for the binuclear Pt(II) complexes (-1255 ppm for $Pt_2(\mu\text{-}Cl)_2Cl_2(H_2O)_2$ and -1363 ppm for $Pt_2(\mu\text{-}Cl)_2Cl_2(C_2H_5OH)_2$) (Entries 9,10, **Supplementary Table 3**) also do not agree with the experiment value (-2680 ppm). Therefore, we could safely rule out the possibility of the divalent state for Pt. The additional calculation on $Pt(II)Cl_2(R^1OR^2)_2$ and $Pt_2(\mu\text{-}Cl)_2Cl_2(R^1OR^2)_2$ supports our previous conclusion of fully reduced state of Pt in the Pt_1@PDMS-PEG.

Supplementary Table 4. DFT structure optimization[a] and calculation of NMR chemical shift and Bader charge of $(R^1OR^2)_2Pt(0)Cl^-_2H^+_2$

Entry	*cis*-divacant octahedral structure and its di-oxygen coordinated structures	^{195}Pt Chemical shift[b] and key structure parameters	Charge on Pt and H (Pt-H) (eV)	Entry	Single-vacant octahedral structure and its CO-coordinated Structures	^{195}Pt and ^{13}C chemical shift[c] and key structure parameters	Charge on Pt (eV)	δ(^{195}Pt) upfield shift (ppm)
**1**	$PtCl^-_2H^+_2$ 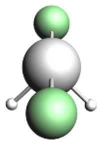	δ(^{195}Pt): -2300 ppm<br><br>Pt–Cl: 2.26 Å<br>Pt–H: 1.52 Å	Pt: 0.52<br>H: 0.14	**8**	$PtCl^-_2H^+_2CO$ 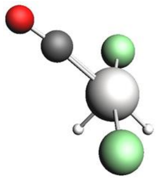	δ(^{195}Pt): -2315 ppm<br>δ(^{13}C): 172.1 ppm<br><br>Pt–Cl: 2.31 Å<br>Pt-C: 2.04 Å	0.61	
**2**	$(H_2O)_2PtCl^-_2H^+_2$ 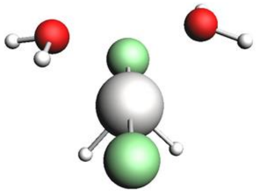	δ(^{195}Pt): -2530 ppm<br><br>Pt–Cl: 2.31 Å<br>Pt–H: 1.53 Å<br>Pt–O: 2.31 Å	Pt: 0.65<br>H: 0.04	**9**	$H_2OPtCl^-_2H^+_2CO$ 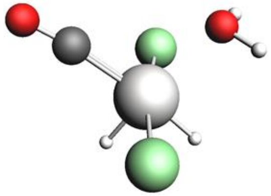	δ(^{195}Pt): -2953 ppm<br>δ(^{13}C): 177.3 ppm<br><br>Pt–Cl: 2.34 Å<br>Pt-C: 2.03 Å<br>Pt-O: 2.31 Å	0.70	423
**3**	$(C_2H_5OH)_2PtCl^-_2H^+_2$ 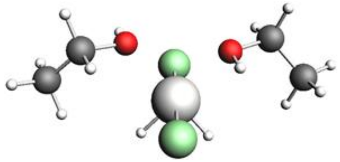	δ(^{195}Pt): -2527 ppm<br><br>Pt–Cl: 2.32 Å<br>Pt–H: 1.53 Å<br>Pt–O: 2.29 Å	Pt: 0.69<br>H: 0.03	**10**	$C_2H_5OHPtCl^-_2H^+_2CO$ 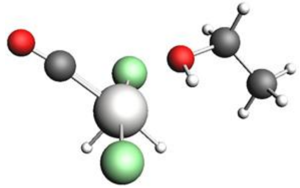	δ(^{195}Pt): -3016 ppm<br>δ(^{13}C): 177.7 ppm<br><br>Pt–Cl: 2.34 Å<br>Pt-C: 2.02 Å<br>Pt-O: 2.29 Å	0.72	489

**4**	$MeO(CH_2)_2OMePtCl^-_2H^+_2$ 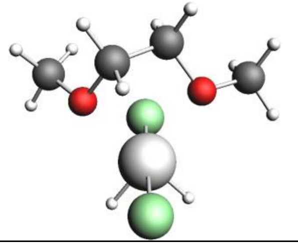	δ(^{195}Pt): -2454 ppm<br><br>Pt–Cl: 2.32 Å<br>Pt–H: 1.53 Å<br>Pt–O: 2.34 Å	Pt: 0.64<br>H: 0.05	**11**	$MeO(CH_2)_2OMePtCl^-_2H^+_2CO$ 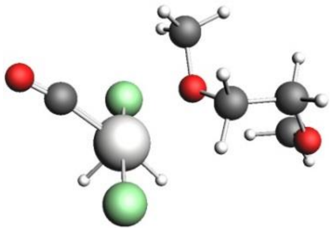	δ(^{195}Pt): -2820 ppm<br>δ(^{13}C): 177.6 ppm<br><br>Pt–Cl: 2.34 Å<br>Pt–C: 2.02 Å<br>Pt–O: 2.34 Å	0.71	366
**5**	$Si(Me)_2(OMe)_2PtCl^-_2H^+_2$ 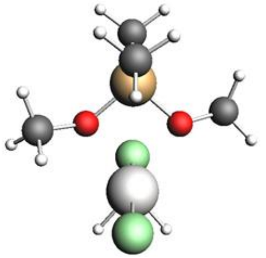	δ(^{195}Pt): -2432 ppm<br><br>Pt–Cl: 2.31 Å<br>Pt–H: 1.53 Å<br>Pt–O: 2.37 Å	Pt: 0.63<br>H: 0.06	**12**	$Si(Me)_2(OMe)_2PtCl^-_2H^+_2CO$ 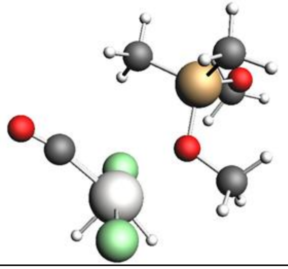	δ(^{195}Pt): -2817 ppm<br>δ(^{13}C): 179.5 ppm<br><br>Pt–Cl: 2.34 Å<br>Pt–C: 2.02 Å<br>Pt–O: 2.33 Å	0.71	385
**6**[d]	$(C_2H_5OH)_2PtCl^-_2(H_3O^+)_2$ 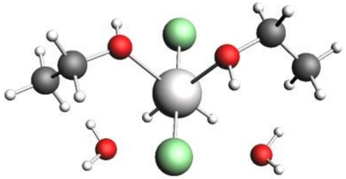	δ(^{195}Pt): -2705 ppm<br>Pt–Cl: 2.32 Å<br>Pt–H: 1.54 Å<br>Pt–O: 2.34 Å<br>O(H_2O)-H($PtCl^-_2H^+_2$):<br>2.28 Å	Pt: 0.61<br>H: 0.08	**13**	CO 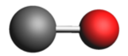	δ(^{13}C): 196.2<br><br>C–O: 1.14 A		
**7**[e]	$(C_2H_5OH)_2PtBr^-_2H^+_2$ 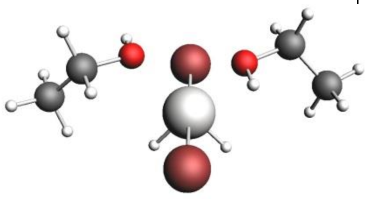	δ(^{195}Pt): -3197 ppm<br><br>Pt–Cl: 2.46 Å<br>Pt–H: 1.54 Å<br>Pt–O: 2.29 Å	H: 0.03	**14**	$Pt(HCl)_2$ 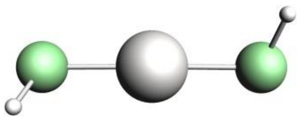	δ(^{195}Pt): -5305 ppm<br><br>Pt–Cl: 2.22 Å<br>Cl–H: 1.31 Å		

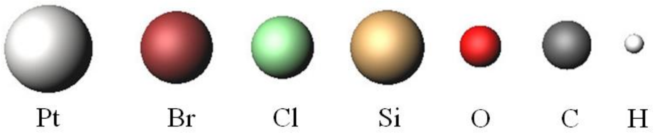	**15**[f]	$Pt(HCl)_2(C_2H_5OH)_2$ 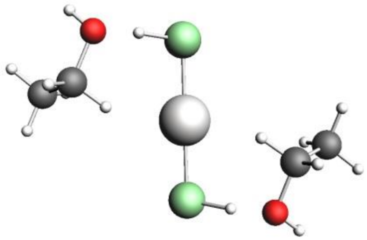	δ(^{195}Pt): -6188 ppm<br><br>Pt–Cl: 2.27 Å<br>Cl–H: 1.43 Å<br>H–O: 1.40 Å		

[a] The chemical structure of PDMS-PEG is given below:

In structure optimization, we began with *cis*-divacant octahedral structure (Entry 1) and single-vacant octahedral structure (Entry 7). The coordinations of these structures to the oxygens of water and ethanol (Entries 2-3, 9-10) were considered. The $MeO(CH_2)_2OMe$ and $Si(Me)_2(OMe)_2$ were used to represent the segment structure of–O–C–C–O in PEG (Entries 4, 11) and -O-Si-O- in PDMS (Entries 5, 12), respectively.

[b] The calculated δ(^{195}Pt) is referenced to $[PtCl_6]^{2-}$. The calculated isotropic nuclear shielding constant σ(^{195}Pt) of this reference is 2005 ppm using the same computational method described in **Methods**.

[c] ^{13}C chemical shift was calculated based on the calculated shielding constant of $Si(Me)_4$ (185 ppm)as a reference compound.

[d] The model $(C_2H_5OH)_2PtCl^-_2(H_3O^+)_2$ was used to evaluate the hydrogen bond interaction, but not limited to this model. With the hydrogen bond interaction, the Pt-H distance increased; the calculated ^{195}Pt NMR chemical shift (-2705 ppm) is also in agreement with the experiment value (-2680 ppm); the charge on H (Pt-H) increased from 0.03 to 0.08 eV.

[e] For the ^{195}Pt NMR calculation of $(R^1OR^2)_2PtBr_2H_2$, ethanol was used as the donating oxygens.

[f] The model $Pt(HCl)_2(C_2H_5OH)_2$ was used to evaluate the effect of Lewis bases on the ^{195}Pt NMR of $Pt(HCl)_2$; ethanol was chosen as a representative of Lewis bases.

For the reduction of H_2PtCl_6 in alcohol and water system, Song *et al.*[9] reported that the reduction of H_2PtCl_6 followed the order of $[PtCl_6]^{2-}$ → $[PtCl_4]^{2-}$ → $[PtCl_2]^{2-}$ → Pt nanoparticles. In our system, The UV-Vis peak (265 nm) corresponding to the ligand-to-metal charge-transfer transition in the $[PtCl_6]^{2-}$ disappeared after reduction, indicating the disappearance of $[PtCl_6]^{2-}$ ions[17]. At the same time, the peak at 220 nm representing $[PtCl_4]^{2-}$ anion didn't appear,

suggesting the absence of $[PtCl_4]^{2-}$ ions[18]. No Pt nanoparticles (as supported by the absence of the characteristic broad peak of Pt nanoparticles between 400 and 800 nm in UV-Vis) were formed.

The chloride ionchromatography analysis indicated a Cl/Pt ratio of 2 in Pt_1@PDMS-PEG. The peak representing the Pt-Cl bond in the Far IR spectrum didn't disappear completely after 3h reduction. Song *et al.*[9] reported the detection of $[Pt(0)Cl_2]^{2-}$ (in the form of $[HPt(0)Cl_2]^{-}$), corresponding to Cl^- coordination to reduced $Pt_1(0)$, in the reduction of H_2PtCl_6 in the methanol-water system. Consistent with the result of Song *et al.*, the Cl/Pt ratio of 2 in this work may be attributed to the weak coordination of Cl^- to $Pt_1(0)$ to form $[PtCl_2]^{2-}$. To validate the reduced state of Pt in this work, the DFT optimized $Pt(II)Cl_2$ structures (Entry 1, **Supplementary Table 3**) was compared with that of $Pt(0)Cl_2H_2$ and $(H_2O)_2Pt(0)Cl_2H_2$ in terms of ^{195}Pt chemical shifts.

For a hypothetical $Pt(II)Cl_2$, the ^{195}Pt NMR chemical shift (60,218 ppm) is out of the reasonable diamagnetic and paramagnetic shift boundaries (Entry 1, **Supplementary Table 3**). For the potential structures of $Pt(II)Cl_2L_2$ (Entries 2-8, **Supplementary Table 3**), the calculated ^{195}Pt NMR chemical shifts are inconsistent with the experimental value (-2680 ppm) for Pt_1@PDMS-PEG. Consistent with the observation of $H^+Pt(0)Cl_2^-$ (Song *et al.*[9]), the core structure of $Pt(0)Cl_2H_2$ consists of two H^+ that balances the charge in $[PtCl_2]^{2-}$ (in an acidic environment). This structure is found to be energetically stable (Entry 1 in **Supplementary Table 4**) by the DFT calculations. In the R^1OR^2 enriched environment (such as PDMS-PEG, ethanol and water), the calculation shows that the $Pt_1(0)$ center of $PtCl^-_2H^+_2$ is favorably coordinated by R^1OR^2. Examples of such structures are given in Entries 2-5 in **Supplementary Table 4**. All these structures produced the calculated ^{195}Pt NMR chemical shifts in agreement with the experimental value of -2680 ppm. When CO is coordinated to $Pt_1(0)$ in $(R^1OR^2)_2PtCl^-_2H^+_2$ to form $(R^1OR^2)PtCl^-_2H^+_2CO$ (Entry 9-12, **Supplementary Table 4**), the corresponding ^{195}Pt NMR chemical shifts are found to be consistent with the experiment value (-3231 ppm) of the CO treated Pt_1@PDMS-PEG. Therefore, the proposed $(R^1OR^2)_2Pt(0)Cl_2H_2$ structure is not only energetically favorable based on the DFT calculations, but also produce the predicted ^{195}Pt chemical shifts that are in agreement with the experimental values.

Song *et al.*[9] showed that the $[Pt(0)Cl_2]^{2-}$ species is an unstable intermediate in the methanol-water solution. It quickly aggregated to form Pt nanoparticles in the methanol-water system. The stability of $(R^1OR^2)_2PtCl^-_2H^+_2$ in our system is facilitated by the protective effect of PDMS-PEG.

We also considered the structures in which two Cl^- are bound directly to Pt(0) while H^+ is bound to Cl^- and between Cl^- and the hydroxyl oxygen of ethanol (Lewis bases) (Entry 14 and 15, **Supplementary Table 4**). Both structures failed to produce the matches to the observed ^{195}Pt chemical shift. It is noted that the structure $(Pt(HCl)_2)$ (Entry 14, ΔE = -2.4 eV) is much less stable than the $Pt(0)Cl^-_2H^+_2$ (Entry 1, ΔE = -4.0 eV).

Supplementary Table 5. DFT structure optimization[a] and calculation of NMR chemical shift of $PtCl_2(CO)_2$ and *cis*-$PtBr_2(CO)_2$.

Entry	Structure	^{195}Pt chemical shift[a]/ppm
1	*trans*-$PtCl_2(CO)_2$ 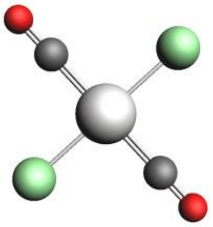	δ(^{195}Pt): -3927ppm<br>Pt–Cl: 2.33 Å<br>Pt–C: 1.92 Å
2	*cis*- $PtCl_2(CO)_2$ 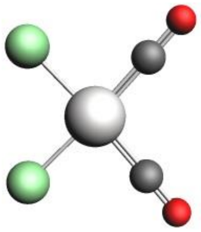	δ(^{195}Pt): -3754 ppm<br>Pt–Cl: 2.30 Å<br>Pt–C: 1.89 Å
3	*cis*- $PtBr_2(CO)_2$ 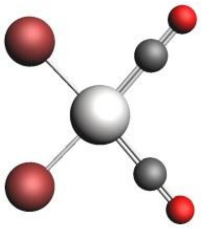	δ(^{195}Pt): -3985 ppm<br>Pt–Br: 2.45 Å<br>Pt–C: 1.89 Å

[a] The calculated δ(^{195}Pt) is referenced to $[PtCl_6]^{2-}$ by the definition of $\delta=(\sigma^{ref}-\sigma)/(1-\sigma^{ref}) \approx (\sigma^{ref}-\sigma)$ with an assumption that $(1-\sigma^{ref}) \approx 1$. The calculated isotropic nuclear shielding constant σ(^{195}Pt) of this reference is 2005 ppm using the same computational method described in "DFT geometry optimization and ^{195}Pt and ^{13}C chemical shift calculation" of **Methods**.

Clearly, the ^{195}Pt chemical shift of the CO di-coordinated Pt(II) structures, *trans*-$PtCl_2(CO)_2$ and *cis*-$PtCl_2(CO)_2$, could not produce predicted chemical shifts in agreement with the experimentally determined value (-3231 ppm, **Figure 2b** in the manuscript).

If the CO treated Pt_1@PDMS-PEG were in the form of *cis*-Pt(II)$Cl_2(CO)_2$, we would anticipate the appearance of the carbonyl bands at 2178 and 2138 cm^{-1} in DRIFT spectrum[33]. However, only one peak at 2084 cm^{-1} appeared for the CO treated Pt_1@PDMS-PEG (**Figure 3b** in the manuscript).

It should be pointed out that, for Pt(II)Cl_2L_2 (L = R^1OR^2), the coordination of two CO (replace L ligand) is energy favorable. Take *cis*-$PtCl_2$-$2C_2H_5OH$ (Entry 5, **Supplementary Table 3**) and *cis*-Pt(II)$Cl_2(CO)_2$ as an example, the first CO replacement has ΔE = -1.38 eV;

and the second CO replacement has ΔE = -0.76 eV. The mono CO coordination to the Pt_1 center in Pt_1@PDMS-PEG indicates the Pt_1 is not in Pt(II) state.

To verify the sensitivity of the DFT calculation, we also calculated the ^{195}Pt chemical shift for *cis*-Pt(II)$Br_2(CO)_2$, and the calculated value of -3985 ppm agrees reasonably with the experimental value of -4243ppm[33].

Supplementary Table 6. Energy dispersive x-ray spectroscopy (EDX) analysis of Pt_1@PDMS-PEG

Elements	Line type	k factor	Adsorption correction	wt%	wt% Sigma
C	K	2.50675	1	80.98	0.63
O	K	1.86867	1	13.32	0.5
Si	K	1.00000	1	3.08	0.19
Cl	K	0.98291	1	0.43	0.08
Pt	L	2.67312	1	2.19	0.35

Note: The data corresponding to Supplementary Figure 5b.

Supplementary Table 7. Comparison of the performance of the catalysts: this work vs literature reported catalysts

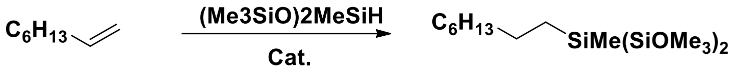

Catalyst		Catalyst (mol%)	Reaction condition	Yield/%	TOF/h^{-1}
This work	Pt_1@PDMS-PEG	5×10^{-4}	50°C, 1 min	99	**1.2×10^{7}**
	PDMS-PEG(blank test)	5×10^{-4}	50°C, 1 min	0	0
Karstedt[38]	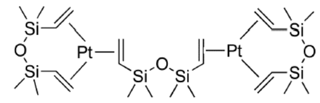	3×10^{-3}	72°C, 15 min	78	**1.4×10^{5}**
		3×10^{-4}	72°C, 240 min	91	7.9×10^{4}
NHC-Pt complex[37,39]	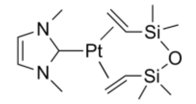	5×10^{-3}	70°C, 200 min	78	4.7×10^{3}
	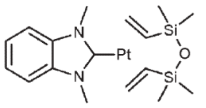	5×10^{-3}	70°C, 200 min	80	4.8×10^{3}
	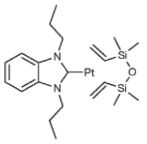	5×10^{-3}	70°C, 100 min	82	**9.8×10^{3}**
	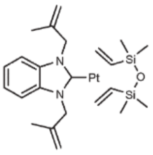	5×10^{-3}	70°C, 150 min	85	6.8×10^{3}
	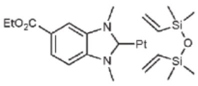	5×10^{-3}	70°C, 200 min	85	5.1×10^{3}
	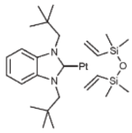	5×10^{-3}	70°C, 150 min	79	6.3×10^{3}
Silylene-Pt complex[38,40]	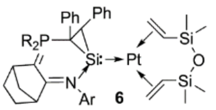	3×10^{-3}	72°C, 20 min	91	9.5×10^{4}
	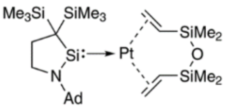	5×10^{-4}	50°C,1h	91	**1.8×10^{5}**

Note: The best performance of the complex in the groups of NHC-Pt and Silyene-Pt is selected to represent each group. Thus the best performance of each group is used as an index in Supplementary Figure 7 to compare with the performance of the catalyst of this work.

Supplementary Table 8. Structure optimization[a] and NMR chemical shift calculation of $(olefin)_1(R^1OR^2)_1PtCl^-_2H^+_2$ using DFT method

Entry	Structures	^{195}Pt Chemical shift[b] and key structure parameters	Charge on Pt (eV)	Entry	Structures	^{195}Pt chemical shift[b] and key structure parameters	Charge (eV)
**1**	$C_3H_6PtCl^-_2H^+_2$ 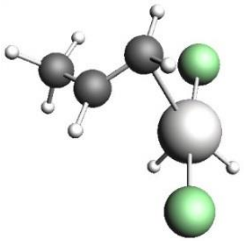	$\delta(^{195}Pt)$: -2071 ppm<br>Pt–Cl: 2.30 Å<br>Pt–C: 2.37 Å	0.51	**5**	$(C_3H_6)_2PtCl^-$ 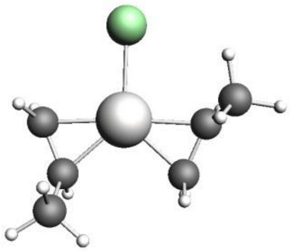	$\delta(^{195}Pt)$: -4334 ppm<br>Pt–Cl: 2.45 Å<br>Pt-C: 2.12 Å	0.24
**2**	$(C_3H_6)(C_2H_5OH)PtCl^-_2H^+_2$ 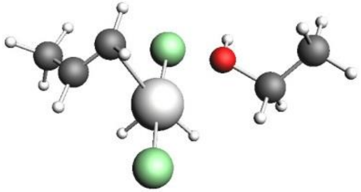	$\delta(^{195}Pt)$: -2688 ppm<br>Pt–Cl: 2.33 Å<br>Pt–C: 2.40 Å<br>Pt–O: 2.31 Å	0.62	**6**	$(C_3H_6)(C_2H_5OH)Pt$ 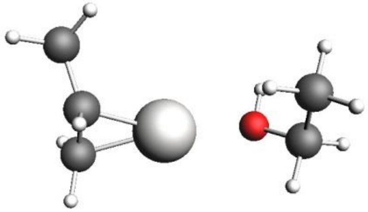	$\delta(^{195}Pt)$: -5804 ppm<br>Pt-C: 2.06 Å<br>Pt-O: 2.13 Å	-0.04
**3**	$(C_3H_6)_2PtCl^-_2H^+_2$ 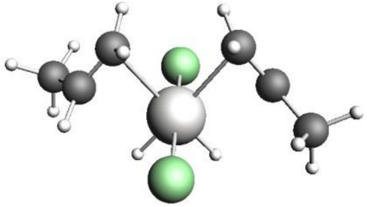	$\delta(^{195}Pt)$: -3746 ppm<br>Pt–Cl: 2.34 Å<br>Pt–C: 2.42 Å	0.52	**7**	C_3H_6Pt 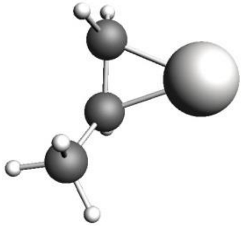	$\delta(^{195}Pt)$: -6999 ppm<br>Pt-C: 2.03 Å	-0.10

**4**	$C_3H_6PtCl^-$ 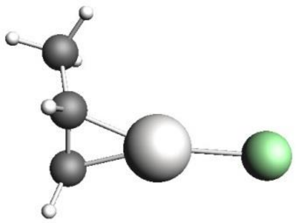	δ(^{195}Pt): -4581 ppm<br><br>Pt–Cl: 2.30 Å<br>Pt-C: 2.07 Å	-0.07	**8**	$(C_3H_6)_2Pt$ 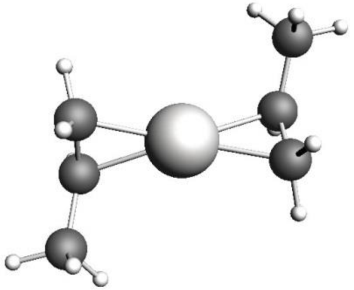	δ(^{195}Pt): -4529 ppm<br><br>Pt–C: 2.14 Å	0.06
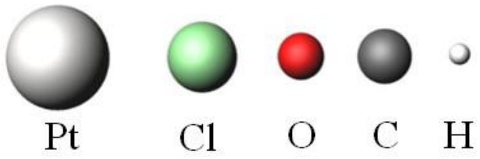				**9**	$(C_3H_6)_3Pt$ 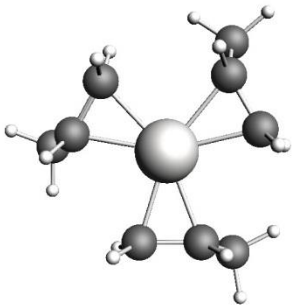	δ(^{195}Pt): -5917 ppm<br><br>Pt–C: 2.17 Å	0.13

[a] For simplicity, $(C_2H_5OH)_2PtCl^-{}_2H^+{}_2$ was chosen as a representative model of $(R^1OR^2)_2PtCl^-{}_2H^+{}_2$ and the propylene was used to represent an olefin. A propylene was added upon the removal of C_2H_5OH, Cl or H correspondingly. All structures were optimized prior to be utilized for the calculation of NMR properties.

[b] The calculated δ(^{195}Pt) is referenced to $[PtCl_6]^{2-}$ as described in in **Methods**.

Supplementary Figures

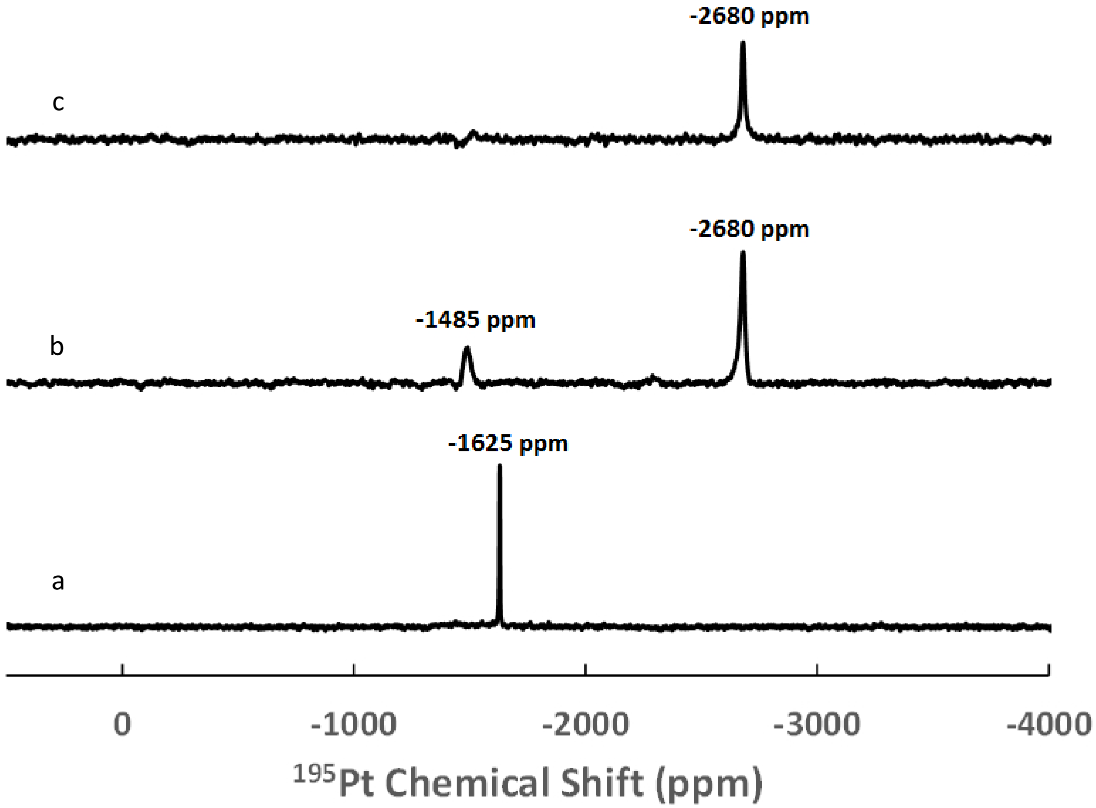

Supplementary Figure 1. ^{195}Pt NMR spectra of K_2PtCl_4 reduction.

(a) The ^{195}Pt NMR chemical shift for K_2PtCl_4 in D_2O is -1625 ppm, closely resembles the reported value[24]. (b) Partial reduction of K_2PtCl_4: add K_2PtCl_4 (76.9 mg or 0.1854 mmol, equivalent to the amount of H_2PtCl_6 used in the 300 ml ethanol-water solution) into PDMS-PEG/ethanol-water (PDMS-PEG: 1.293 g, ethanol-water solution: 10 ml, V(ethanol)/V(water) = 9/1), stirred for **5** min before evacuation of ethanol and water at 40°C. Two ^{195}Pt NMR peaks (-1485 ppm and -2680 ppm) were observed, indicating the partial reduction of K_2PtCl_4. (c) Full reduction of K_2PtCl_4: the same mixture was stirred for **30** min followed evacuation of ethanol and water at 40°C. Only one ^{195}Pt NMR peak at -2680 ppm was observed, indicating the full reduction of K_2PtCl_4.

The reduction of $[PtCl_6]^{2-}$ takes two steps (**Supplementary Table 2**). Since the electric potential from $[PtCl_6]^{2-}$ to $[PtCl_4]^{2-}$ (0.68 eV) is lower than that from $[PtCl_4]^{2-}$ to Pt^0 (0.755 eV), the reduction of $[PtCl_4]^{2-}$ would be faster than that of the reduction of $[PtCl_6]^{2-}$ to $[PtCl_4]^{2-}$. **Supplementary Figure 1b** and **1c** demonstrate that ^{195}Pt chemical shifts of Pt (II) and $Pt_1(0)$ in PDMS-PEG matrix are -1485 ppm and -2680 ppm, respectively. The absence of the peak in the range between -1485 and -1625 ppm in

Pt_1@PDMS-PEG indicates full reduction of Pt_1.

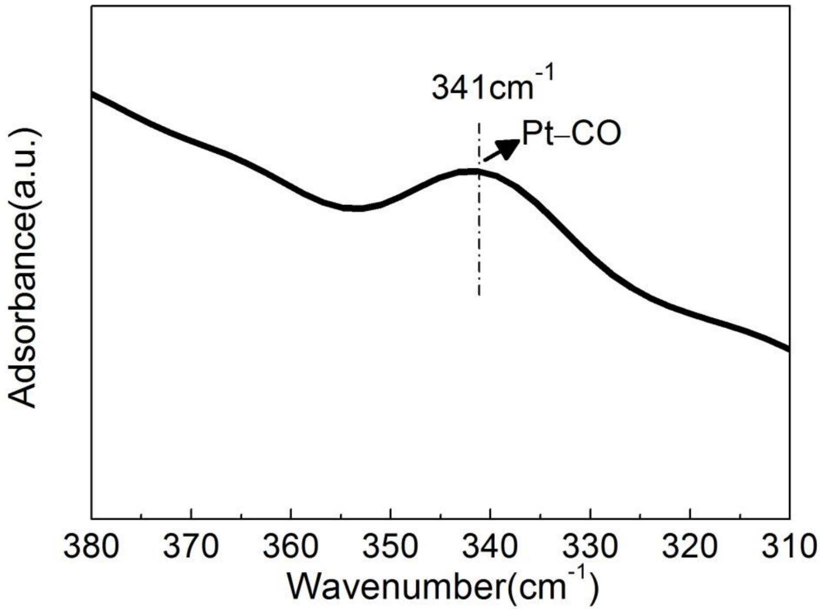

Supplementary Figure 2. Far infrared spectrum of the Pt_1-CO bond after CO was introduced into the stock solution (see Methods).

The appearance of the peak at 341 cm^{-1} corresponds to the formation of Pt_1-CO bond.

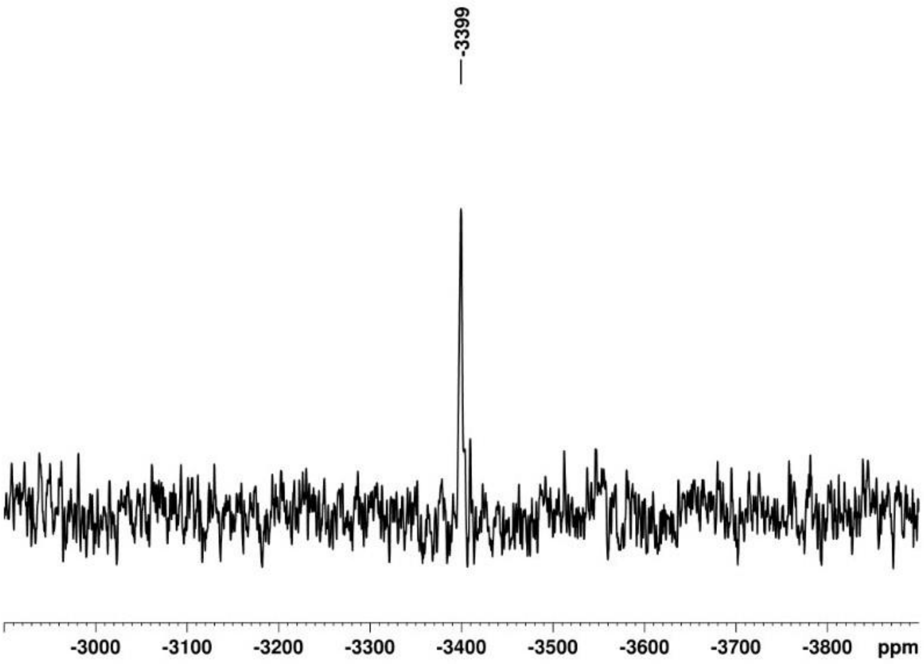

Supplementary Figure 3. ^{195}Pt NMR of HBr exchanged Pt_1@PDMS-PEG.

Excess HBr aqueous solution was added into the 10 ml concentrated Pt_1@PDMS-PEG solution (150 ml concentrated to 10 ml). Stir at room temperature for 12 h before ^{195}Pt NMR measurement.

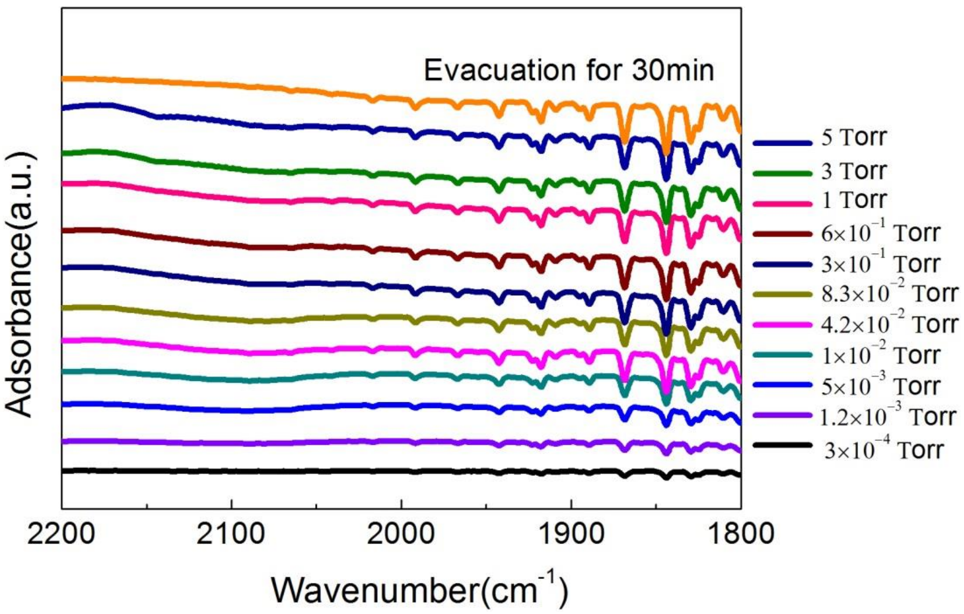

Supplementary Figure 4. ***In situ*** **DRIFT spectra of CO absorption as a function of CO pressure on PDMS-PEG/SiO_2.**

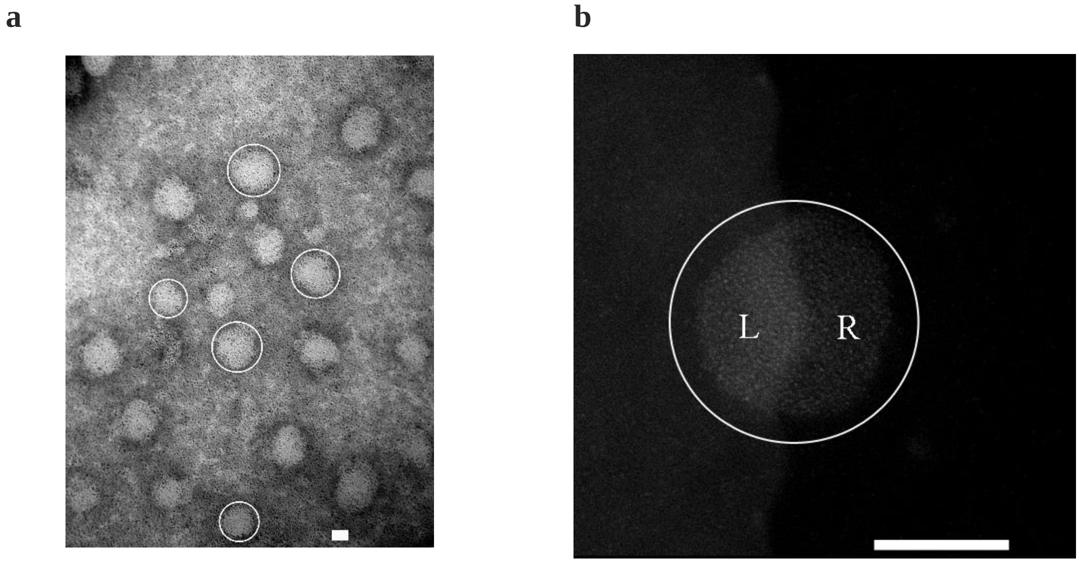

Supplementary Figure 5. TEM (a) and STEM (b) images of Pt_1@PDMS-PEG.

20 nm scale bar for a, and 50 nm scale bar for b. For TEM characterization, phosphotungstic acid was used as the staining reagent. From the TEM image, fairly unilamellar vesicles with a narrow distribution (30-80 nm) were obtained. The STEM image of a Pt_1@PDMS-PEG vesicle with the circular area displays a bright region on the left (L) and a less bright region on the right (R). The left side (L) of the vesicle is located at the junction of a copper grid part of the sample holder, and the right side (R) corresponds to the other part on ultrathin carbon film of the sample holder. The average diameter of Pt_1@PDMS-PEG structure in STEM was identical to that observed under TEM. Because the elements in PDMS-PEG are indistinguishable under STEM, and Pt is the only heavy element in the Pt_1@PDMS-PEG solution, the lighted circular areas, both (L) and (R), can be ascribed to $Pt_1(0)$ atoms encapsulated in the newly formed PDMS-PEG vesicles, and energy dispersive x-ray spectroscopy (EDX) analysis also confirmed the existence of Pt on the vesicle (Supplementary Table 6). Further magnification of the image was not available due to formation of Pt nanoparticles from the aggregation of $Pt_1(0)$ single atoms, induced by the high energy electron beams (For STEM, no phosphotungstic acid was used).

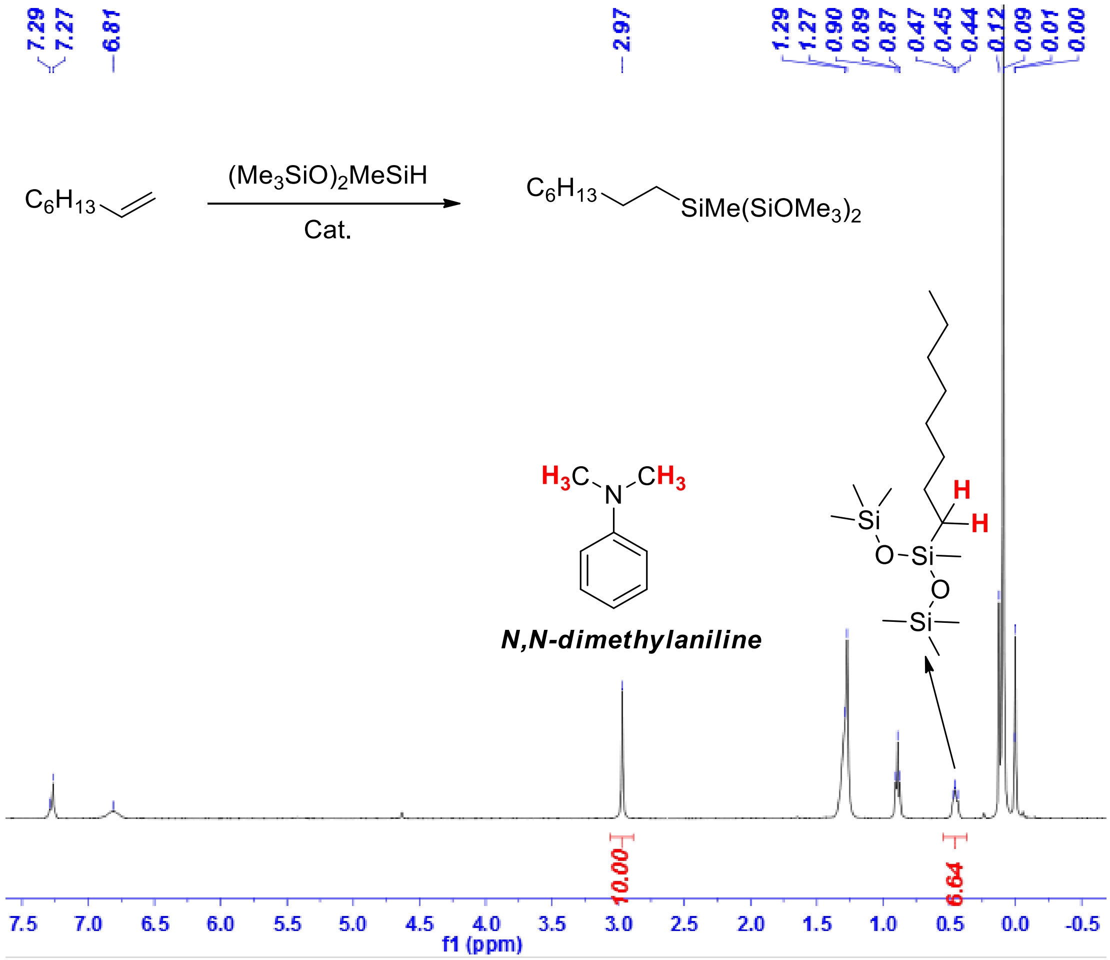

Supplementary Figure 6. ^{1}**H NMR spectrum of reaction mixture.**

Performed on 4 mmol scale. $(Me_3SiO)_2MeSiH$/1-octene = 1.1 (mol/mol), temperature = 50 °C, [Pt_1@PDMS-PEG] = 5 × 10 $^{-4}$ mol% (based on 1-octene), no solvent. The conversion and selectivity were determined by ^{1}H NMR analysis using *N,N*-dimethylaniline as internal standard.

Notes on 1-octene conversion and terminal product (**T**) yield and selectivity of the hydrosilytion reaction between 1-octene and $(Me_3SiO)_2MeSiH$:

To calculate the conversion of 1-octene and selectivity of product, we first defined the following parameters:

P: NMR peak integral; *N*: number of protons; *n*: number of mmoles.

The *N,N*-dimethylaniline was used as the internal standard, so we have:

$$n_{1-\text{octene}} = n_{N,N-dimethylaniline} \frac{N_{N,N-dimethylaniline}}{N_{1-\text{octene}}} \frac{P_{1-\text{octene}}}{P_{N,N-dimethylaniline}}$$

Using similar equation, $n_{\boldsymbol{T}}$ was also calculated.

$$n_{\boldsymbol{T}} = n_{N,N-dimethylaniline} \frac{N_{N,N-dimethylaniline}}{N_{T-product}} \frac{P_{T-product}}{P_{N,N-dimethylaniline}}$$

For the hydrosilylation of 1-octene and 1,1,1,3,5,5,5-heptamethyltrisiloxane at 50 °C with 1 min,

we have:

$n_{N,N-dimethylaniline}$= 1.98 mmol (0.2400g)

$P_{N,N-dimethylaniline}$= 10.00

$P_{1-octene}$ = 0

$P_{\boldsymbol{T}}$ = 6.64

The calculated molar quantities (in mmol) of the unconverted 1-octene and product **T** are:

$$n_{T\ product} = n_{N,N-dimethylaniline} \frac{N_{N,N-dimethylaniline}}{N_{\boldsymbol{T}}} \frac{P_{\boldsymbol{T}}}{P_{N,N-dimethylaniline}}$$

$$= 1.98 \times \frac{6}{2} \times \frac{6.64}{10} = 3.94\ mmol$$

and

$$n_{1-octene} = n_{N,N-dimethylaniline} \frac{N_{N,N-dimethylaniline}}{N_{1-octene}} \frac{P_{1-octene}}{P_{N,N-dimethylaniline}}$$

$$= 1.98 \times \frac{6}{2} \times \frac{0}{10} = 0.0$$

The conversion, therefore, can be calculated as:

$$Conversion = \left(1 - \frac{n_{1-octene}}{n_{1-octene,0}}\right) \times 100\ \%$$

$$= \left(1 - \frac{0}{4}\right) \times 100\ \% = 100\ \%$$

$n_{\ 1-octene,0}$ $(4\ mmol)$ is the initial 1-octene substrate in mmol.

The selectivity of **T** is calculated as:

$$Selectivity = \left(\frac{n_{\boldsymbol{T}}}{n_{1-octene,0} - n_{1-octene}}\right) \times 100\ \%$$

$$= \left(\frac{3.94}{4-0}\right) \times 100\ \% = 99\%$$

The yield of **T** is calculated as:

$$Yield = Conversion \times Selectivity \times 100\ \%$$

$$= 99\%$$

The turnover frequency (TOF) is defined as the number of moles of olefin converted per mole of $Pt_1(0)$ per unit of time, expressed in h^{-1} :

$$TOF\ = \frac{n_{1-octene,0} \times yield_T}{n_{Pt}} \times \frac{1}{t}$$

n_{Pt} is the number of mmole $Pt_1(0)$ used in the hydrosilytion and *t* is reaction time.

Thus, the TOF can be calculated as

$$TOF = \frac{n_{1-octene,0} \times yield_T}{n_{Pt}} \times \frac{1}{t}$$

$$= \left(\frac{3.94}{2 \times 10^{-5}}\right) \times \frac{1}{1}$$

$$= 2 \times 10^5 min^{-1}$$

$$= 1.2 \times 10^7 h^{-1}$$

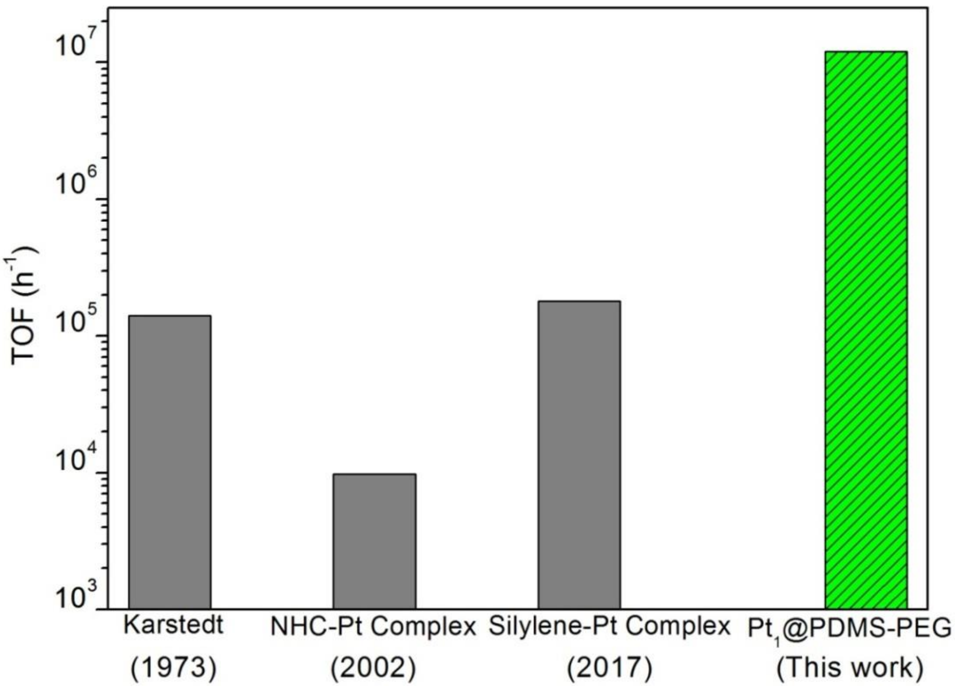

Supplementary Figure 7. Comparison of the performance of the catalysts: this work vs literature reported catalyst.

The TOF numbers chosen for comparison were the ones representing the best performance in each catalyst group. See Supplementary Table 7 for details. (Pt_1@PDMS-PEG is the catalyst of this work)

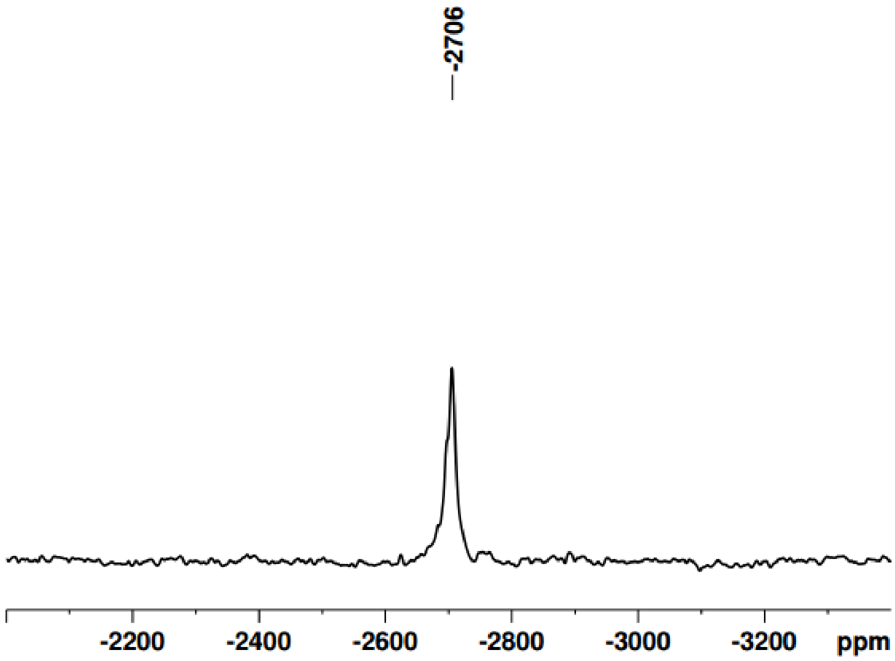

Supplementary Figure 8. ^{195}Pt NMR spectrum of (olefin)(R^1OR^2)$PtCl^-_2H^+_2$.

Concentrated 150ml Pt_1@PDMS-PEG solution to 20ml. Added 0.0990g 1-octene (10 times equivalent to Pt_1(0)). Stirred for 24 h at 40°C and followed by further concentrating the solution to about 3ml. The sample was collected in a glove box under N_2 prior to the ^{195}Pt NMR measurement.

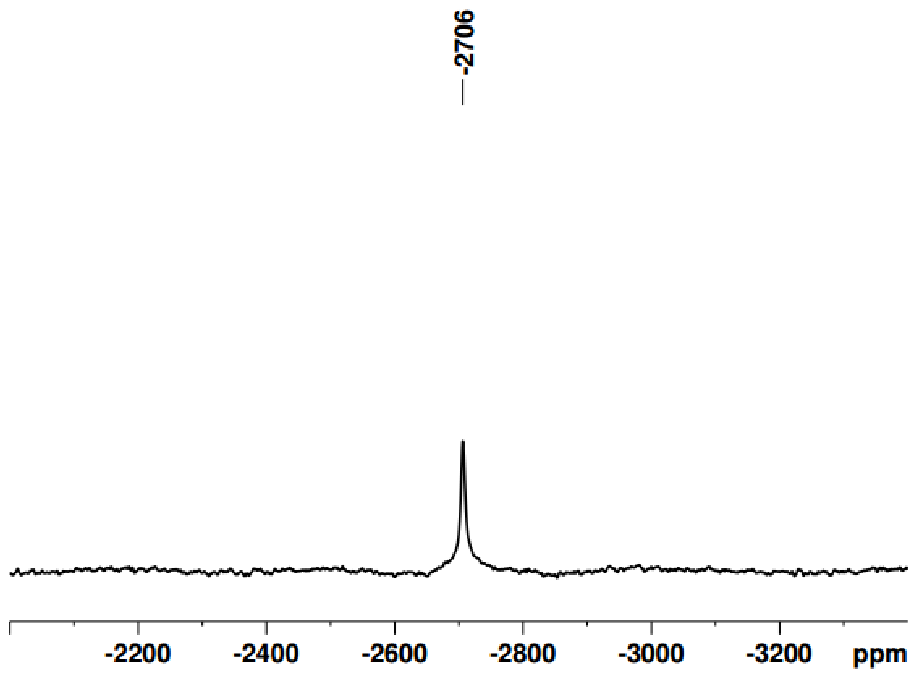

Supplementary Figure 9. ^{195}Pt NMR spectrum of the catalyst after hydrosilylation (with 1-octene coordinated).

Sample preparation: Concentrate 150ml Pt_1@PDMS-PEG/ethanol-water to 5ml, and add ethanol to 150ml, and add 4.95g 1-octene (500 times equivalent to $Pt_1(0)$) into the system, and stir for 10min. Add 5.88g $(Me_3SiO)_2MeSiH$ (300 times equivalent to $Pt_1(0)$) into the system and stir for 1h at 50°C. Further concentrate the solution to about 3ml and collect the sample in a glove box under N_2. Because of the excess amount of olefin (1-octene), the $Pt_1(0)$ maintained the (olefin)$(R^1OR^2)PtCl^-_2H^+_2$ state (^{195}Pt chemical shift), not $(R^1OR^2)_2PtCl^-_2H^+_2$ state.

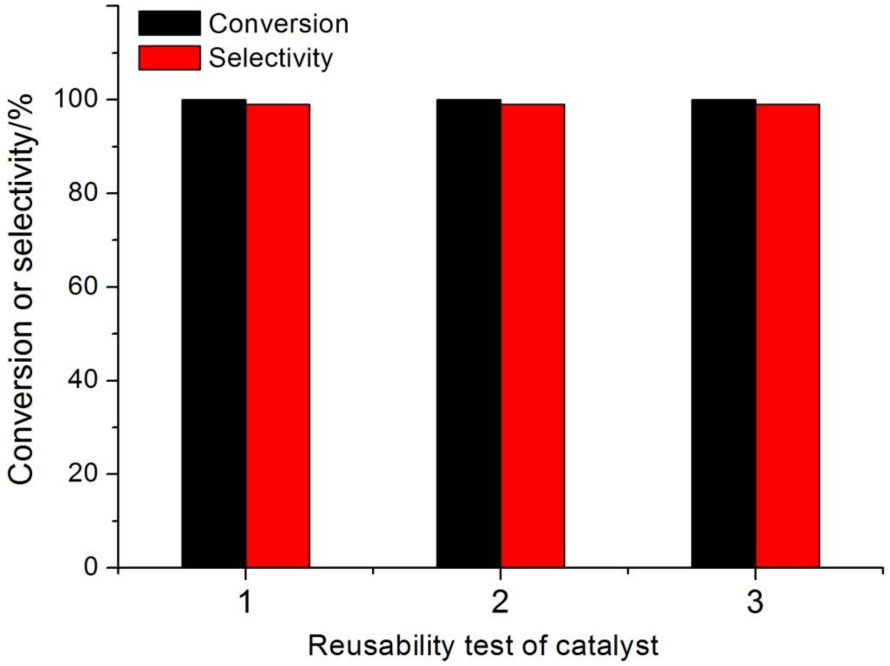

Supplementary Figure 10. Conversion/selectivity vs. number of catalyst reuse of hydrosilylation between 1-octene and $(Me_3SiO)_2MeSiH$ ($n_{1\text{-octene}}$: n_{silane} = 1:1).

The reaction conditions were the same as that used for a fresh catalyst (see **Methods**).
