# Supplementary material for: Genesis of electron deficient Pt1(0) in PDMS-PEG aggregates
Source: Nat Commun. 2019 Mar 1;10:996. doi: 10.1038/s41467-019-08804-y (PMC6397160; doi:10.1038/s41467-019-08804-y)
Supplement: Supplementary file 2 — Peer Review File [file 41467_2019_8804_MOESM2_ESM.pdf]

Reviewers' comments:

Reviewer #1 (Remarks to the Author):

This paper describes the easy method to prepare the single atom catalyst of Pt. In this method, the use of polydimethylsiloxane-polyethylene glycol (PDMS-PEG) copolymer, which weakly supports the Pt atom, as an amphiphilic solvating agent successfully prevent the aggregation of Pt metals. Formation of the Pt1 species is supported by various spectroscopic data. For example, the full conversion of the starting platinum chlorides were confirmed by UV-vis and 195Pt NMR. Dispersion of Pt atoms were also supported by both UV and NMR. The reduced oxidation state of Pt is supported by XPS. DFT calculation was also performed to support these experimental data. However, although I am not an expert of this field, the identification seems to be not sufficient considering previous reports often measured EXAFS, EXANES, STEM etc, which directly support the formation of Pt1 species.
It is of interest the developed Pt1 catalysts exhibit higher activity in the olefin hydrosilylation reaction. In this point, the easy preparation method is a great advantage. To enhance the importance of this work, it is recommended to show additional data on the stability and the reusability of the catalyst.

Reviewer #2 (Remarks to the Author):

The manuscript by Bai, Zhang and co-workers reports on the synthesis of "electron deficient "Pt1" in PDMS-PEG" aggregates.
It is claimed that in this work the authors generate "discrete platinum atoms Pt1 by reducing H2PtCl6 in PDMS-PEG aggregates. Later the authors write that the Pt1 species exists as a pseudo octahedral structure of the type (ROR)2PtCl2H2. The Pt1@PDMS-PEG showed ultra-high activity in olefin hydrosilylation.
In the following introduction, the authors point the interest of making single reduced metal atoms rather than metal clusters. They then state that "reduced metal atoms in their conventional organometallic complexes are strongly bonded by electron rich ligands". What is the meaning of "conventional organometallic complex"?
Moreover, unlike what is written by the authors, in fact reduced metal centers are classically stabilized by electron accepting ligands, such as CO they are using later on.
They claim that being able to make "discrete reduced metal atoms, distinguishable from organometallic complexes, in liquid media may lead to precise synthesis of new materials at atomic scale". They are apparently confused with the definition of a metal complex, which is a molecule made of a metal center (in any formal oxidation state: positive, 0 or even negative, 0 being the "reduced metal atom" they refer to) and ligands (any type: neutral L type, anionic X type). There is no such thing as a discrete reduced metal atom in a liquid medium, free of any coordination by a ligand. These would agglomerate and make bulk material. In fact, coordination of solvent molecules acting as ligands to the metal center is very common in organometallic chemistry.
In agreement, they propose that the Pt1 reduced species is (ROR)2PtCl2H2, which is nothing else than a complex.
Therefore, the whole introduction should be modified in depth.

The authors have carefully studied the reduction of the Pt(IV) starting complex by EtOH in the presence of the PDMS-PEG polymer in EtOH/H2O mixture. They state on page 3 line 80, that "complete reduction of the Pt ions" is seen after 3h at 105°C. I do not understand what the authors mean by "loss of coulombic Pt-Cl bonds". There is still a signal in far IR for the Pt-Cl bond, and they show by ion chromatography that Cl/Pt ratio is 2. It is shown that Pt clusters are not formed.
The question at this point is the nature of the Pt species. The authors claim that the absence of signals near 0 and -1617 ppm "indicates full reduction of platinum". It is not correct. It shows the efficient reduction of Pt(IV), but not the full reduction to Pt(0). Indeed, the Karstedt complex, a Pt(0) complex, features a signal at much higher field (-6130 ppm). They observe a signal at -2680 ppm. 195Pt NMR

chemical shifts are indeed really sensitive to the electronic environment of the Pt center and therefore to the ligands. Notably, NMR signals for Pt(II) complexes have been observed at much higher field than -2680 ppm (see for ex JOMC 2001, 622, 180).
The authors state that in Karstedt complex, "the platinum atoms are highly electron rich due to strongly electron donating by 1,3-divinyl-1,1,3,3-tetramethyldisiloxane". This is not correct. The Pt center is electron rich because it is a formal Pt(0), but the dvtms ligands are both sigma donor and pi acceptors via the two double bonds. In fact, it is because the ligand is (good pi) acceptor that the Pt(0) complex is stable and isolable.
In order to further characterize the Pt species Pt1, the authors made a very interesting experiment using labelled and non labelled CO. They observed a significant upfield shift (-3231ppm), proving coordination of CO to the Pt center. Interestingly, the Pt NMR proves the coordination of only one CO ligand to the Pt center.
Note that in the JOMC paper cited above, $PtCl_2(CO)_2$ is said to be colorless, just like the solution obtained upon reduction of H_2PtCl_6 here. Moreover, for example cis-$PtBr_2(CO)_2$, a defined Pt(II) complex, has a chemical shift of -4243 ppm which is a much lower field than the -3231 ppm for the Pt1 species.
The authors propose a structure $(ROR)_2PtCl_2H_2$ for Pt1, based on DFT calculations of Pt chemical shifts. However the structures proposed are Pt(IV) complexes with formally 2 Cl- and 2 H- ligands. This is not consistent with the NMR and XPS data, which both point to lower oxidation state at Pt. There is no experimental evidence for the 2 H ligands at Pt. The authors should record the proton coupled 195Pt NMR spectrum. If the two H are indeed bound to the Pt center, a triplet should be observed.
Alternatively, Pt-H are also very well characterized by 1H NMR spectroscopy at high field. The corresponding signal should present the expected Pt satellites.
It is difficult to trust the NMR chemical shifts of 195 Pt predicted by DFT for proposed structures, when the prediction of the chemical shift of CO is -11.2 ppm, compared to real chemical shift at ca 185 ppm.
To me the NMR and XPS data is more consistent with either monometallic square planar $Pt(Cl)_2(L)_2$, (Pt(II) center) or bimetallic Pt complexes with bridging Cl ligands, (L)(Cl)Pt(mu2-Cl)2Pt(Cl)(L) with L being the PDMS "shell". Several bridging Cl Pt dimers are known in the literature.
In these types of complexes, the poorly donating "PDMS" ligand (via O) would be readily displaced by CO.
The Pt1 species being a Pt(II) complex would also be consistent with Cl to Br exchange using HBr.
Finally, it is very doubtful that Pt1 is a discrete Pt(0) complex because the reactivity with octane was done in the presence of $CHCl_3$, a very oxidizing solvent for low oxidation state Pt center. On the other hand Pt(II) complexes are stable toward $CHCl_3$.
To me a very important question is not addressed: what is the size and shape of the PDMS-PEG "vesicle" in the EtOH/H_2O mixture. Does it accept more than one Pt complex? DOSY NMR experiments should be carried out to answer this question.

In conclusion for the synthesis/characterization of the Pt1 species, the data is not consistent with the proposed structure.

The application in hydrosilylation is on the other hand truly outstanding, with much better TOFs and TONs than known systems, which could on its own warrant publication in Nat. Comm.
The blank tests need to be carried out.
Indeed, if the PDMS has been obtained via hydrosilylation, some Pt might remain in the polymer.
It is claimed that after the catalytic run, the exact same complex is seen by NMR (identical chemical shift). This is rather surprising, as it means that olefin bound Pt complex "(olefin)(R1OR2)PtCl2H2" exists as the resting state in the catalytic process, although it reacts very fast with the silane. Have the authors tried the independent reaction of the complex with silane? What does it lead to?
Finally, if the complex is intact after a first catalytic run, reloading and recycling experiments should be performed.

In conclusion, I believe that the generation of a highly active Pt complex in PDMS-PEG for hydrosilylation of alkenes is of high interest. However, I am not convinced at all by the structure proposed by the authors, and several additional experiments should be carried out. Comparison with data in the literature for Pt(II) complexes should also be done. Moreover, the introduction needs to be modified in depth.
For these reasons, I recommend rejection of the article, but I believe it would make a nice contribution to Nat. Comm once the points raised are addressed.

Reviewers' comments:

Reviewer #1 (Remarks to the Author):

This paper describes the easy method to prepare the single atom catalyst of Pt. In this method, the use of polydimethylsiloxane-polyethylene glycol (PDMS-PEG) copolymer, which weakly supports the Pt atom, as an amphiphilic solvating agent successfully prevent the aggregation of Pt metals. Formation of the Pt1 species is supported by various spectroscopic data. For example, the full conversion of the starting platinum chlorides were confirmed by UV-vis and 195Pt NMR. Dispersion of Pt atoms were also supported by both UV and NMR. The reduced oxidation state of Pt is supported by XPS. DFT calculation was also performed to support these experimental data.
However, although I am not an expert of this field, the identification seems to be not sufficient considering previous reports often measured EXAFS, EXANES, STEM etc, which directly support the formation of Pt1 species.

Response:

The TEM and the EDX analysis in STEM have been used to characterize the morphology of Pt_1@PDMS-PEG (**Supplementary Figure 5a**) and the distribution of Pt in the vesicles (**Supplementary Figure 5b** and **Supplementary Table 5**).

The new results and discussions are included in the revised manuscript: "The morphology of Pt_1@PDMS-PEG solution was characterized by transmission electron microscopy (TEM). Fairly unilamellar vesicles with a narrow distribution (30-80 nm, Supplementary Figure 5a) were formed[34]. The energy-dispersive X-ray spectroscopy analysis in a scanning transmission electron microscope (STEM) revealed the existence of Pt in the vesicles (Supplementary Figure 5b and Supplementary Table 5). It should be noted that the Pt atoms tend to agglomerate under intense electron beam or X-ray radiation, making TEM, STEM and synchrotron X-ray measurements infeasible in providing unambiguous evidence of discrete Pt atoms in our system[35]."

(a) (b)

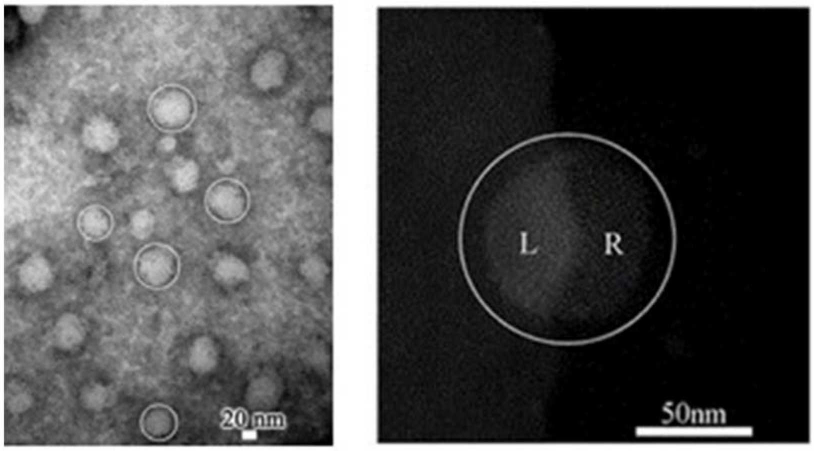

Supplementary Figure 5. TEM (a) and STEM (b) images of Pt_1@PDMS-PEG. For TEM characterization, phosphotungstic acid was used as the staining reagent. From the TEM image, fairly unilamellar vesicles with a narrow distribution (30-80 nm) were obtained. The STEM image of a Pt_1@PDMS-PEG vesicle with the circular area displays a bright region on the left (L) and a less bright region on the right (R). The left side (L) of the vesicle is located at the junction of a copper grid part of the sample holder, and the right side (R) corresponds to the other part on ultrathin carbon film of the sample holder. The average diameter of Pt_1@PDMS-PEG structure in STEM was identical to that observed under TEM. Because the elements in PDMS-PEG are indistinguishable under STEM, and Pt is the only heavy element in the Pt_1@PDMS-PEG solution, the lighted circular areas, both (L) and (R), can be ascribed to Pt_1 atoms encapsulated in the newly formed PDMS-PEG vesicles, and energy dispersive x-ray spectroscopy (EDX) analysis also confirmed the existence of Pt on the vesicle (Supplementary Table 5). Further magnification of the image was not available due to formation of Pt nanoparticles from the aggregation of Pt_1 single atoms, induced by the high energy electron beams (For STEM, no phosphotungstic acid was used).

It is of interest the developed Pt1 catalysts exhibit higher activity in the olefin hydrosilylation reaction. In this point, the easy preparation method is a great advantage. To enhance the importance of this work, it is recommended to show additional data on the stability and the reusability of the catalyst.

Response:

We tested used catalyst for three times. The results are given in **Supplementary Figure 10**. A discussion is given in the revised manuscript: "Moreover, through three recycled uses, Pt_1@PDMS-PEG maintained high activity without noticeable deactivation in 1-octene hydrosilylation with $(Me_3SiO)_2MeSiH$ (Supplementary Figure 10). The color of the reused Pt_1@PDMS-PEG solution remained clear throughout the reuses."

The procedure is given in **Methods:** "After the hydrosilylation reaction between 1-octene and $(Me_3SiO)_2MeSiH$ (1-octene: 0.4488 g, $(Me_3SiO)_2MeSiH$: 0.89 g) finished, the solution was centrifuged. 0.6694 g reaction mixture was taken out from the upper solution, and analyzed by ^1H NMR. The remaining solution (0.6694 g) was used for the subsequent hydrosilylation reaction. For the reuse test, 0.4488 g 1-octene and 0.89 g $(Me_3SiO)_2MeSiH$ was added. After reaction, solution was centrifuged. 1.3388 g (equal to the amount of added reactants) reaction mixture was taken out from the upper solution, and analyzed by ^1H NMR, while the remaining solution (0.6694 g) was used for the subsequent hydrosilylation reaction. For the following reuses, the added amounts of 1-octene and $(Me_3SiO)_2MeSiH$ were always 0.4488 g and 0.89 g, respectively. After each reaction, the amount of reaction mixture taken out for ^1H NMR from the upper solution after centrifugation was always 1.3388 g (equal to the amount of added reactants), and the remaining solution was kept at 0.6694 g for the subsequent hydrosilylation reaction."

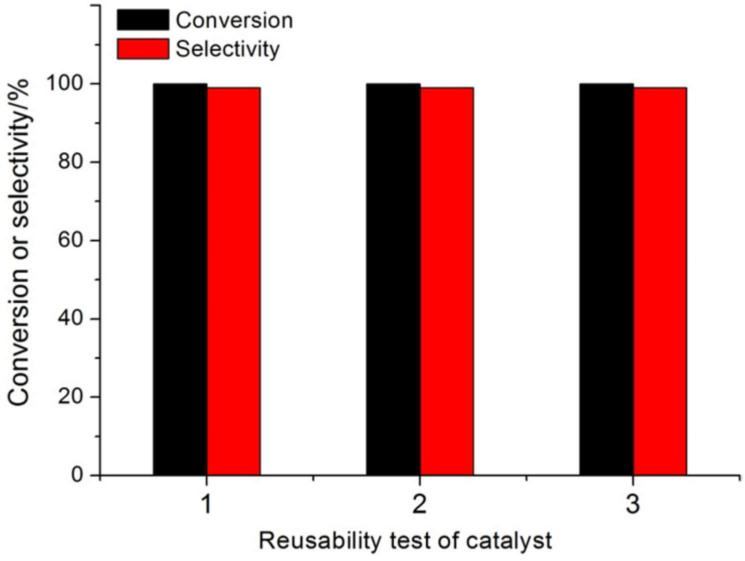

Supplementary Figure 10. Conversion/selectivity vs. number of catalyst reuse of hydrosilylation between 1-octene and $(Me_3SiO)_2MeSiH$ ($n_{1\text{-octene}} : n_{silane}$ = 1:1). The reaction conditions were the same as that used for a fresh catalyst (see **Methods**)

Reviewer #2 (Remarks to the Author):

The manuscript by Bai, Zhang and co-workers reports on the synthesis of "electron deficient "Pt1" in PDMS-PEG" aggregates.
It is claimed that in this work the authors generate "discrete platinum atoms Pt1 by reducing H_2PtCl_6 *in PDMS-PEG aggregates. Later the authors write that the Pt1 species exists as a pseudo octahedral structure of the type* $(ROR)_2PtCl_2H_2$*. The Pt1@PDMS-PEG showed ultra-high activity in olefin hydrosilylation.*

In the following introduction, the authors point the interest of making single reduced metal atoms rather than metal clusters. They then state that "reduced metal atoms in their conventional organometallic complexes are strongly bonded by electron rich ligands". What is the meaning of "conventional organometallic complex"? Moreover, unlike what is written by the authors, in fact reduced metal centers are classically stabilized by electron accepting ligands, such as CO they are using later on.
They claim that being able to make "discrete reduced metal atoms, distinguishable from organometallic complexes, in liquid media may lead to precise synthesis of new materials at atomic scale". They are apparently confused with the definition of a metal complex, which is a molecule made of a metal center (in any formal oxidation state: positive, 0 or even negative, 0 being the "reduced metal atom" they refer to) and ligands (any type: neutral L type, anionic X type). There is no such thing as a discrete reduced metal atom in a liquid medium, free of any coordination by a ligand. These would agglomerate and make bulk material. In fact, coordination of solvent molecules acting as ligands to the metal center is very common in organometallic chemistry. In agreement, they propose that the Pt1 reduced species is $(ROR)_2PtCl_2H_2$*, which is nothing else than a complex. Therefore, the whole introduction should be modified in depth.*

Response: We thank the reviewer's comments, and the term of "conventional organometallic complex" has been removed from the revised manuscript. Instead, we emphasize that $(ROR)_2PtCl_2H_2$ has a weakly coordinated mononuclear electron deficient Pt_1. Detailed characteristics of the $(ROR)_2PtCl_2H_2$ structure have been fully discussed in the revised manuscript. What is important for the Pt_1 structure of this work is that the H is protic, instead of the hydride as in conventional metal-hydride bond. More evidence and further discussion are given in the revised manuscript to clarify the differences. More details are also given below in responses to the specific questions.

To me the NMR and XPS data is more consistent with either monometallic square planar Pt(Cl)2(L)2, (Pt(II) center) or bimetallic Pt complexes with bridging Cl ligands, (L)(Cl)Pt(mu2-Cl)2Pt(Cl)(L) with L being the PDMS "shell". Several bridging Cl Pt dimers are known in the literature. In these types of complexes, the poorly donating "PDMS" ligand (via

O) would be readily displaced by CO. The Pt1 species being a Pt(II) complex would also be consistent with Cl to Br exchange using HBr.

Response: To further confirm Pt_1 was in reduced state, rather than Pt (II), we monitored the reduction process of $[PtCl_4]^{2-}$ in PDMS-PEG using ^{195}Pt NMR and the results are shown in **Supplementary Figure 1** :

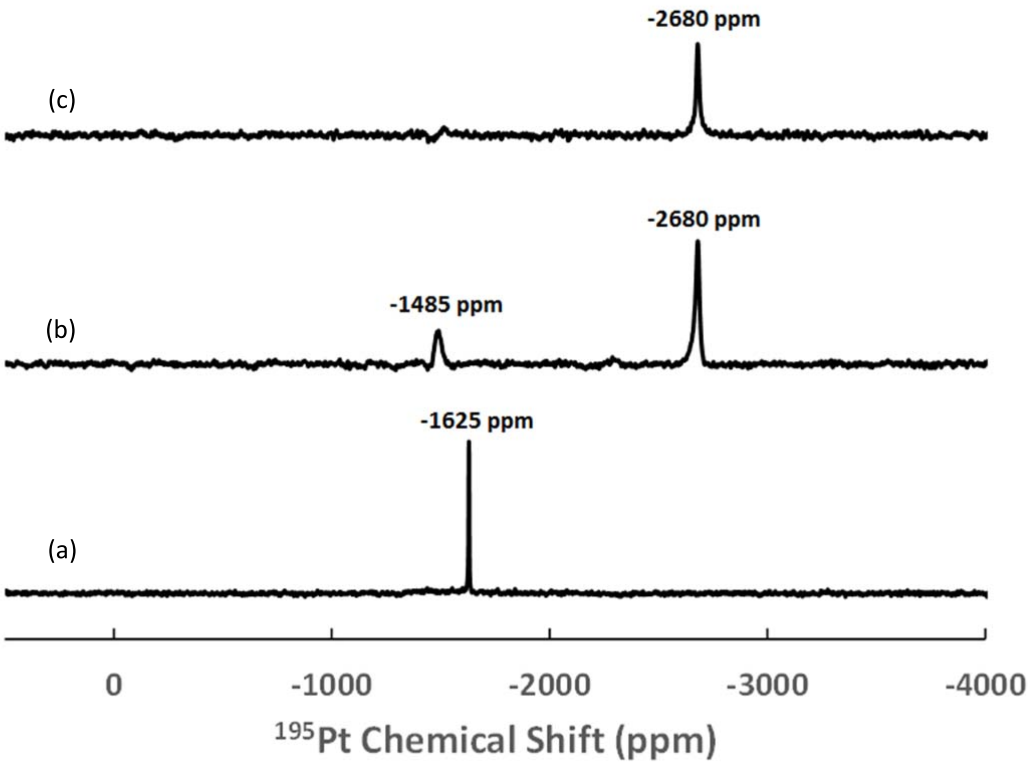

Supplementary Figure 1. ^{195}Pt NMR spectra of K_2PtCl_4 reduction. (a) The ^{195}Pt NMR chemical shift for K_2PtCl_4 in D_2O is -1625 ppm, closely resembles the reported value[24]. (b) Partial reduction of K_2PtCl_4: add K_2PtCl_4 (76.9 mg or 0.1854 mmol, equivalent to the amount of H_2PtCl_6 used in the 300 ml ethanol-water solution) into PDMS-PEG/ethanol-water (PDMS-PEG: 1.293 g, ethanol-water solution: 10 ml, V(ethanol)/V(water) = 9/1), stirred for **5** min before evacuation of ethanol and water at 40°C. Two ^{195}Pt NMR peaks (-1485 ppm and -2680 ppm) were observed, indicating the partial reduction of K_2PtCl_4. (c) Full reduction of K_2PtCl_4: the same mixture was stirred for **30** min followed evacuation of ethanol and water at 40°C. Only one ^{195}Pt NMR peak at -2680 ppm was observed, indicating the full reduction of K_2PtCl_4.

Supplementary Equation 1. Reduction of H_2PtCl_6

$$E^{\Theta}/V$$

$$PtCl_6^{2-} + CH_3CH_2OH \longrightarrow PtCl_4^{2-} + CH_3CHO + 2H^+ + 2Cl^- \qquad 0.68$$

$$PtCl_4^{2-} + CH_3CH_2OH \longrightarrow Pt_1^0 + CH_3CHO + 2H^+ + 4Cl^- \qquad 0.755$$

We have added the discussion in Supplementary Materials (below the caption of **Supplementary Figure 1**) "The reduction of $[PtCl_6]^{2-}$ takes two steps (**Supplementary Equation 1**). Since the electric potential from $[PtCl_6]^{2-}$ to $[PtCl_4]^{2-}$ (0.68 eV) is lower than that from $[PtCl_4]^{2-}$ to Pt^0 (0.755 eV), the reduction of $[PtCl_4]^{2-}$ would be faster than that of the reduction of $[PtCl_6]^{2-}$ to $[PtCl_4]^{2-}$. **Supplementary Figure 1b** and **1c** demonstrate that ^{195}Pt chemical shifts of Pt (II) and $Pt_1(0)$ in PDMS-PEG matrix are -1485 ppm and -2680 ppm, respectively. The absence of the peak in the range between -1485 and -1625 ppm in Pt_1@PDMS-PEG indicates full reduction of Pt_1."

The related results are added to revised manuscript "This conclusion has been independently verified by the reduction of $[PtCl_4]^{2-}$ in PDMS-PEG monitored by ^{195}Pt NMR (Supplementary Figure 1)."

Computationally, several possible structures of $PtCl_2(R^1OR^2)_2$ and $Pt_2(\mu\text{-}Cl)_2Cl_2(R^1OR^2)_2$, in which the Pt is in Pt(II) state, are optimized and the results are compared with the $(R^1OR^2)_2PtCl_2H_2$ in which the Pt is in reduced state as proposed in this work. The new computational results for $Pt(II)Cl_2(R^1OR^2)_2$ and $Pt_2(\mu\text{-}Cl)_2Cl_2(R^1OR^2)_2$ were added to **Supplementary Table 2** (The following table is provided for the sake of convenience):

Supplementary Table 2. DFT structure optimization[a] and calculation of NMR chemical shift of $PtCl_2L_2$ and $Pt_2(\mu\text{-}Cl)_2Cl_2L_2$ (L= R^1OR^2).

Entry	Structures	^{195}Pt Chemical shift[b] and key structure parameters
1	Pt(II)Cl_2 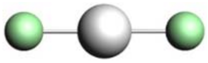	δ(^{195}Pt): 60218 ppm<br><br>Pt–Cl: 2.16 Å
2	*trans*-$PtCl_2$-2H_2O 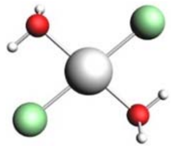	δ(^{195}Pt): -1427 ppm<br><br>Pt–Cl: 2.31 Å<br>Pt–O: 2.06 Å
3	*cis*-$PtCl_2$-2C_2H_5OH 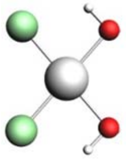	δ(^{195}Pt): -1387 ppm<br><br>Pt–Cl: 2.26 Å<br>Pt–O: 2.14 Å
4	*trans*-$PtCl_2$-2C_2H_5OH 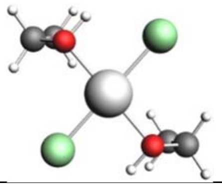	δ(^{195}Pt): - 1569 ppm<br><br>Pt–Cl: 2.31 Å<br>Pt–O: 2.05 Å
5	*cis*-$PtCl_2$-2C_2H_5OH 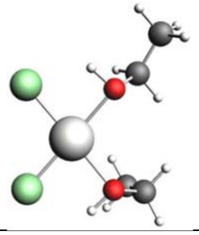	δ(^{195}Pt): -1572 ppm<br><br>Pt–Cl: 2.27 Å<br>Pt–O: 2.13 Å
6	*trans*-$PtCl_2$-2CH_3OCH_3 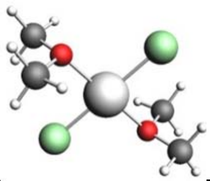	δ(^{195}Pt): -1442 ppm<br><br>Pt–Cl: 2.32 Å<br>Pt–O: 2.06 Å
7	*cis*-$PtCl_2$-2CH_3OCH_3 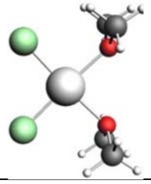	δ(^{195}Pt): -1439 ppm<br><br>Pt–Cl: 2.26 Å<br>Pt–O: 2.15 Å

8	$PtCl_2$-$SiO_4(CH_3)_4$ 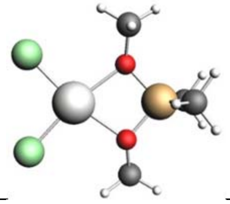	δ(^{195}Pt): -1076 ppm<br><br>Pt–Cl: 2.25 Å<br>Pt–O: 2.17 Å
9[c]	$Pt_2(\mu\text{-}Cl)_2Cl_2(H_2O)_2$ 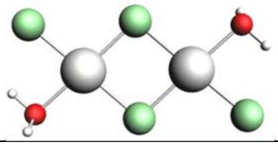	δ(^{195}Pt): -1255 ppm<br><br>Pt–Cl: 2.28/2.35 Å<br>Pt–O: 2.11 Å
10[c]	$Pt_2(\mu\text{-}Cl)_2Cl_2(C_2H_5OH)_2$ 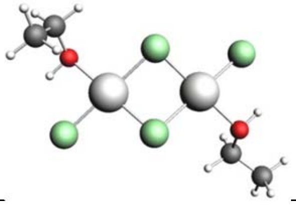	δ(^{195}Pt): -1363 ppm<br><br>Pt–Cl: 2.29/2.35 Å<br>Pt–O: 2.09 Å
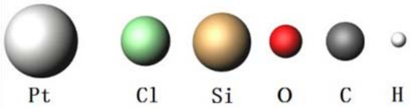		

[a] In structure optimization, the coordinations in these structures by the oxygens of water and ethanol (Entries 2-5) were considered. The MeOMe and $Si(Me)_2(OMe)_2$ were used to represent the segment structure of–O–C–C–O in PEG (Entries 6, 7) and O-Si-O in PDMS (Entries 8), respectively.

[b] The calculated δ(^{195}Pt) is referenced to $[PtCl_6]^{2-}$ by the definition of $\delta=(\sigma^{ref}-\sigma)/(1-\sigma^{ref}) \approx (\sigma^{ref}-\sigma)$ with an assumption that $(1-\sigma^{ref}) \approx 1$. The calculated isotropic nuclear shielding constant σ(^{195}Pt) of this reference is 2005 ppm using the same computational method described in "DFT geometry optimization and ^{195}Pt and ^{13}C chemical shift calculation" of **Methods**.

[c] For the $Pt_2(\mu\text{-}Cl)_2Cl_2L_2$, only ethanol and water were considered.

We added the discussion in the Supplementary Materials (attached to **Supplementary Table 2**): "The calculated ^{195}Pt chemical shift for $Pt(II)Cl_2(R^1OR^2)_2$ (Entries 2-8, **Supplementary Table 2**) are in a range of -1076 to -1572 ppm, in reasonable agreement with the experimental ^{195}Pt chemical shift of Pt(II) ($[PtCl_4]^{2-}$) in the range of -1485 and -1617 ppm[24], that is distinctively different from the experiment value (-2680 ppm) of Pt_1@PDMD-PEG. The calculated ^{195}Pt chemical shifts for the binuclear Pt(II) complexes (-1255 ppm for $Pt_2(\mu\text{-}Cl)_2Cl_2(H_2O)_2$ and -1363 ppm for $Pt_2(\mu\text{-}Cl)_2Cl_2(C_2H_5OH)_2$) (Entries 9,10, **Supplementary Table 2**) also do not agree with the experiment value (-2680 ppm). Therefore, we could safely rule out the possibility of the divalent state for Pt. The additional calculation on

$Pt(II)Cl_2(R^1OR^2)_2$ and $Pt_2(\mu\text{-}Cl)_2Cl_2(R^1OR^2)_2$ supports our previous conclusion of fully reduced state of Pt in the Pt_1@PDMS-PEG. ”

The corresponding results and discussion are added in the revised manuscript “^{195}Pt NMR calculation of Pt(II) in $PtCl_2L_2$ or binuclear $Pt_2(\mu\text{-}Cl)_2Cl_2L_2$ complexes with bridging Cl ligands (Supplementary Table 2) also supports the reduced state of Pt_1 center [25]. ”

The authors have carefully studied the reduction of the Pt(IV) starting complex by EtOH in the presence of the PDMS-PEG polymer in EtOH/H_2O mixture. They state on page 3 line 80, that “complete reduction of the Pt ions” is seen after 3h at 105°C. I do not understand what the authors mean by “loss of coulombic Pt-Cl bonds”. There is still a signal in far IR for the Pt-Cl bond, and they show by ion chromatography that Cl/Pt ratio is 2. It is shown that Pt clusters are not formed

Response: We added the discussion in the Supplementary Materials (attached to Supplementary Table 3): “For the reduction of H_2PtCl_6 in alcohol and water system, Song *et al.* [9] reported that the reduction of H_2PtCl_6 followed the order of $[PtCl_6]^{2-}$ → $[PtCl_4]^{2-}$ → $[PtCl_2]^{2-}$ → Pt nanoparticles. In our system, The UV-Vis peak (265 nm) corresponding to the ligand-to-metal charge-transfer transition in the $[PtCl_6]^{2-}$ disappeared after reduction, indicating the disappearance of $[PtCl_6]^{2-}$ ions[17]. At the same time, the peak at 220 nm representing $[PtCl_4]^{2-}$ anion didn’t appear, suggesting the absence of $[PtCl_4]^{2-}$ ions[18]. No Pt nanoparticles (as supported by the absence of the characteristic broad peak of Pt nanoparticles between 400 and 800 nm in UV-Vis) were formed.

The chloride ionchromatography analysis indicated a Cl/Pt ratio of 2 in Pt_1@PDMS-PEG. The peak representing the Pt-Cl bond in the Far IR spectrum didn’t disappear completely after 3h reduction. Song *et al.*[9] reported the detection of $[Pt(0)Cl_2]^{2-}$ (in the form of $HPt(0)Cl_2^-$), corresponding to Cl^- coordination to reduced Pt_1, in the reduction of H_2PtCl_6 in the methanol-water system. Consistent with the result of Song *et al.*, the Cl/Pt ratio of 2 in this work may be attributed to the weak coordination of Cl^- to Pt_1^0 to form $PtCl_2^{2-}$. To validate the reduced state of Pt in this work, the DFT optimized $Pt(II)Cl_2$ structures (Entry 1, **Supplementary Table 2**) was compared with that of $Pt(0)Cl_2H_2$ and $(H_2O)_2Pt(0)Cl_2H_2$ in terms of ^{195}Pt chemical shifts.

For a hypothetical Pt(II)Cl_2, the ^{195}Pt NMR chemical shift (60,218 ppm) is out of the reasonable diamagnetic and paramagnetic shift boundaries (Entry 1, **Supplementary Table 2**). For the potential structures of Pt(II)Cl_2L_2 (Entries 2-8, **Supplementary Table 2**), the calculated ^{195}Pt NMR chemical shifts are inconsistent with the experimental value (-2680 ppm) for Pt_1@PDMS-PEG. Consistent with the observation of H^+Pt(0)Cl_2^- (Song *et al.*[9]), the core structure of Pt(0)Cl_2H_2 consists of two H^+ that balances the charge in $PtCl_2^{2-}$ (in an acidic environment). This structure is found to be energetically stable (Entry 1 in **Supplementary Table 3**) by the DFT calculations. In the R^1OR^2 enriched environment (such as PDMS-PEG, ethanol and water), the calculation shows that the Pt_1 center of $PtCl_2H_2$ is favorably coordinated by R^1OR^2. Examples of such structures are given in Entries 2-5 in **Supplementary Table 3**. All these structures produced the calculated ^{195}Pt NMR chemical shifts in agreement with the experimental value of -2680 ppm. When CO is coordinated to Pt_1 in $(R^1OR^2)_2PtCl_2H_2$ to form $(R^1OR^2)PtCl_2H_2CO$ (Entry 9-12, **Supplementary Table 3**), the corresponding ^{195}Pt NMR chemical shifts are found to be consistent with the experiment value (-3231 ppm) of the CO treated Pt_1@PDMS-PEG. Therefore, the proposed $(R^1OR^2)_2$Pt(0)Cl_2H_2 structure is not only energetically favorable based on the DFT calculations, but also produce the predicted ^{195}Pt chemical shifts that are in agreement with the experimental values.

Song *et al.*[9] showed that the Pt(0)Cl_2^{2-} species is an unstable intermediate in the methanol-water solution. It quickly aggregated to form Pt nanoparticles in the methanol-water system. The stability of $(R^1OR^2)_2PtCl_2H_2$ in our system is facilitated by the protective effect of PDMS-PEG."

The question at this point is the nature of the Pt species. The authors claim that the absence of signals near 0 and -1617 ppm "indicates full reduction of platinum". It is not correct. It shows the efficient reduction of Pt(IV), but not the full reduction to Pt(0). Indeed, the Karstedt complex, a Pt(0) complex, features a signal at much higher field (-6130 ppm). They observe a signal at -2680 ppm. ^{195}Pt NMR chemical shifts are indeed really sensitive to the electronic environment of the Pt center and therefore to the ligands. Notably, NMR signals for Pt(II) complexes have been observed at much higher field than -2680 ppm (see for ex JOMC 2001, 622, 180).

Response: Indeed, ^{195}Pt NMR chemical shifts are very sensitive to the electronic environment of the Pt center, which makes ^{195}Pt NMR an excellent probe for oxidation state and coordination environment. It is well documented that ^{195}Pt NMR chemical shifts of H_2PtCl_6 and $[PtCl_4]^{2-}$ are around 0 ppm and -1617 ppm, respectively (Inorganic chemistry, 2004, 43, 1421-1428). The ^{195}Pt NMR chemical shifts for Pt(II) in PDMS-PEG (**Supplementary Figure 1b**) is -1485 ppm (this work). The reduction process of $[PtCl_4]^{2}$ in PDMS-PEG matrix investigated by ^{195}Pt NMR (**Supplementary Figure 1)** unambiguously demonstrates that Pt_1 in PDMS-PEG is in reduced state with a characteristic resonance at -2680 ppm.

The authors state that in Karstedt complex, "the platinum atoms are highly electron rich due to strongly electron donating by 1,3-divinyl-1,1,3,3-tetramethyldisiloxane". This is not correct. The Pt center is electron rich because it is a formal Pt(0), but the dvtms ligands are both sigma donor and pi acceptors via the two double bonds. In fact, it is because the ligand is (good pi) acceptor that the Pt(0) complex is stable and isolable.

Response: We thank the reviewer's comments and have removed the related statements about electron rich in the Pt center of Karstedt complex. We added the discussion in the revised manuscript: "The Bader charge (~0.6 eV) on the Pt_1 center in $(R^1OR^2)_2PtCl_2H_2$ (Entries 2-5, Supplementary Table 3) is much higher than the charge (0.13 eV) for Pt in the tri-olefin coordinated complex (Entry 9, Supplementary Table 7), representing Karstedt Pt catalyst. ^{195}Pt chemical shift of the Pt_1@PDMS-PEG is about 3450 ppm down-field shifted to that of the Karstedt Pt catalyst[42], also in conformity with the high electron deficiency of the Pt_1@PDMS-PEG catalyst."

In order to further characterize the Pt species Pt1, the authors made a very interesting experiment using labelled and non labelled CO. They observed a significant upfield shift (-3231ppm), proving coordination of CO to the Pt center. Interestingly, the Pt NMR proves the coordination of only one CO ligand to the Pt center. Note that in the JOMC paper cited above, PtCl2(CO)2 is said to be colorless, just like the solution obtained upon reduction of H2PtCl6 here. Moreover, for example cis-PtBr2(CO)2, a defined Pt(II) complex, has a chemical shift of -4243 ppm which is a much lower field than the -3231 ppm for the Pt1 species.

Response: The ^{195}Pt and ^{13}C NMR results indicate only one CO is coordinated to the Pt_1 center when Pt_1@PDMS-PEG was exposed to CO. In our work, the ^{195}Pt NMR, ^{13}C NMR and

CO infrared spectra all consistently showed the preference of mono-carbonyl coordination to the Pt_1 center in Pt_1@PDMS-PEG. To rationalize the domination of mono-carbonyl coordination to the Pt_1 center, we added a statement to the revised manuscript, based on our new calculation, as: "… replacing the first R^1OR^2 group with CO is energetically favored (ΔE = -0.18 eV). However, further replacement of the remaining R^1OR^2 with a CO resulted in negligible energy difference (ΔE = -0.04eV), consistent with fact that only one CO binds to Pt_1 atom. "

The calculated ^{195}Pt chemical shifts for the hypothetical structures of *trans*-Pt(II)$Cl_2(CO)_2$, *cis*-Pt(II)$Cl_2(CO)_2$ and *cis*-Pt(II)$Br_2(CO)_2$, where two CO's are coordinated, are given in the **Supplementary Table 4** (A table is provided below for the sake of convenience):

Supplementary Table 4. DFT structure optimization[a] and calculation of NMR chemical shift of $PtCl_2(CO)_2$ and cis-$PtBr_2(CO)_2$.

Structure	^{195}Pt chemical shift[a]/ppm
trans-$PtCl_2(CO)_2$ 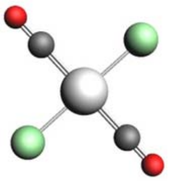	-3927
cis- $PtCl_2(CO)_2$ 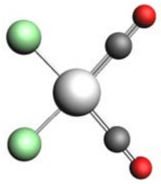	-3754
cis- $PtBr_2(CO)_2$ 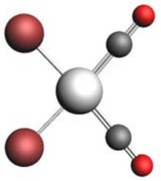	-3985

[a] The calculated $\delta(^{195}Pt)$ is referenced to $[PtCl_6]^{2-}$ by the definition of $\delta=(\sigma^{ref}-\sigma)/(1-\sigma^{ref}) \approx (\sigma^{ref}-\sigma)$ with an assumption that $(1-\sigma^{ref}) \approx 1$. The calculated isotropic nuclear shielding constant $\sigma(^{195}Pt)$ of this reference is 2005 ppm using the same computational method described in "DFT geometry optimization and ^{195}Pt and ^{13}C chemical shift calculation" of **Methods**.

We added the discussion in the Supplementary Materials (below **Supplementary Table 4**) "Clearly, the ^{195}Pt chemical shift of the CO di-coordinated Pt(II) structures, *trans*-$PtCl_2(CO)_2$

and *cis*-$PtCl_2(CO)_2$, could not produce predicted chemical shifts in agreement with the experimentally determined value (-3231 ppm, **Figure 2b** in the manuscript).

If the CO treated Pt_1@PDMS-PEG were in the form of *cis*-Pt(II)$Cl_2(CO)_2$, we would anticipate the appearance of the carbonyl bands at 2178 and 2138 cm^{-1} in DRIFT spectrum[33]. However, only one peak at 2084 cm^{-1} appeared for the CO treated Pt_1@PDMS-PEG (**Figure 3b** in the manuscript).

It should be pointed out that, for Pt(II)Cl_2L_2 (L = R^1OR^2), the coordination of two CO (replace L ligand) is energy favorable. Take *cis*-$PtCl_2$-2C_2H_5OH (Entry 5, **Supplementary Table 2**) and *cis*-Pt(II)$Cl_2(CO)_2$ as an example, the first CO replacement has ΔE = -1.38 eV; and the second CO replacement has ΔE = -0.76 eV. The mono CO coordination to the Pt_1 center in Pt_1@PDMS-PEG indicates the Pt_1 is not in Pt(II) state.

To verify the sensitivity of the DFT calculation, we also calculated the ^{195}Pt chemical shift for *cis*-Pt(II)$Br_2(CO)_2$, and the calculated value of -3985 ppm agrees reasonably with the experimental value of -4243ppm[33]."

With the new results on Pt(II), we added discussion about the DFT calculation in the revised Manuscript: "DFT calculation also shows that di-coordinated Pt(II) structures, *trans*-Pt(II)$Cl_2(CO)_2$ and *cis*-Pt(II)$Cl_2(CO)_2$, could not produce predicted chemical shifts in agreement with the experimentally observed value -3231 ppm (Supplementary Table 4). In addition, unlike (R^1OR^2)Pt(0)Cl_2H_2, the formation of dicarbonyl complex, Pt(II)$Cl_2(CO)_2$, from Pt(II)Cl_2(R^1OR^2) is energetically favorable by the DFT calculations (Supplementary Table 4)."

Summary discussion about the DRIFT results was added to the revised manuscript: "the characteristic peaks at 2178 and 2138 cm^{-1} corresponding to the *cis*-Pt(II)$Cl_2(CO)_2$ were absent[33]"

The authors propose a structure $(ROR)_2PtCl_2H_2$ for Pt_1, based on DFT calculations of Pt chemical shifts. However the structures proposed are Pt(IV) complexes with formally 2 Cl- and 2 H- ligands. This is not consistent with the NMR and XPS data, which both point to lower oxidation state at Pt. There is no experimental evidence for the 2 H ligands at Pt. The authors should record the proton coupled ^{195}Pt NMR spectrum. If the two H are indeed bound to the Pt center, a triplet should be observed. Alternatively, Pt-H are also very well characterized by 1H NMR spectroscopy at high field. The corresponding signal should present the expected Pt satellites.

Response: The reviewer raised an excellent point about ^{1}H-^{195}Pt coupling, which led to an in-depth investigation of the issue. Our explanation based on further computational has been added in the revised version: "The Bader charge of hydrogen is positive for all optimized $(R^1OR^2)_2PtCl_2H_2$ structures (Entries 2-5, Supplementary Table 3), suggesting the H in $(R^1OR^2)_2PtCl_2H_2$ is similar to labile proton rather than to hydride in nature. In the presence of abundant protons such as in residual H_2O and OH groups of the solvent, the protic H in the $(R^1OR^2)_2PtCl_2H_2$ could be readily hydrated, e.g. H_3O^+. The calculated ^{195}Pt chemical shift (-2705 ppm) of $(R^1OR^2)_2Pt(0)Cl_2(H_3O^+)_2$ (Entry 6, Supplementary Table 3) is in an excellent agreement with the experimental shift (-2680 ppm), suggesting that $(R^1OR^2)_2Pt(0)Cl_2(H_3O^+)_2$ may exist as a hydrated form of $(R^1OR^2)_2PtCl_2H_2$ in the system. This calculated result is a further validation of protic H, rather than a hydride. The absence of $\boldsymbol{J}(^{195}Pt$-$^{1}H)$ spin-spin couplings in the ^{195}Pt spectra may be explained by the rapid chemical exchanges between the protic H in $(R^1OR^2)_2PtCl_2H_2$ and abundant labile protons of water and hydroxyl protons in the system. Furthermore, the protic H in the $(R^1OR^2)_2PtCl_2H_2$ is consistent with a recent report that showed HCl acted as a weak ligand in the stabilization of Ir nanoparticles with the H in protic state[30]."

It is difficult to trust the NMR chemical shifts of ^{195}Pt predicted by DFT for proposed structures, when the prediction of the chemical shift of CO is -11.2 ppm, compared to real chemical shift at ca 185 ppm.

Response: We simply used the absolute ^{13}C NMR shielding constant (-11.2 ppm) in the original version, instead of the chemical shift, for CO in the text. To address the reviewer's concern, we calculated the ^{13}C chemical shieldings for $Si(Me)_4$ (as a reference), the CO in gas phase, and the $(R^1OR^2)PtCl_2H_2CO$. The ^{13}C NMR chemical shifts were given in Entries 9-12, **Supplementary Table 3**. The calculated shifts of (R^1OR^2) $PtCl_2H_2CO$ is about 24 ppm up-field shifted with respective to the gas phase CO, in the same trend of ~35 ppm up-field shift observed experimentally. We have converted all shielding constants to chemical shift values in the revised manuscript and Supplementary Materials. The expression in the revised Manuscript "In concert with the experimental observation, the density functional theory

(DFT) calculation of ^{13}C NMR chemical shift also shows a 24.1 ppm up-field shift from the gas phase CO to Pt•••C=O (Entries 8, 13 in Supplementary Table 3)..”

Finally, it is very doubtful that Pt_1 is a discrete Pt(0) complex because the reactivity with octane was done in the presence of CHCl3, a very oxidizing solvent for low oxidation state Pt center. On the other hand Pt(II) complexes are stable toward CHCl3.

Response: We have stated that no solvent was used in the hydrosilylation reaction in the section of “General Procedure for the hydrosilylation of 1-octene catalyzed by Pt_1@PDMS-PEG” in **Methods**.

Only an adequate amount of $CDCl_3$ was used during the ^{195}Pt NMR characterization of the 1-octene treated Pt_1@PDMS-PEG to enhance the solubility. If Pt_1@PDMS-PEG is oxidized by small amount of $CHCl_3$, one would expect a change in the ^{195}Pt chemical shift to down field after 1-octene treated Pt_1@PDMS-PEG. In fact, a ^{195}Pt NMR shift of -2706 ppm was observed n, very close in value to that of the Pt_1@PDMS-PEG (-2680 ppm), indicating Pt(0) exists throughout the processes.

To me a very important question is not addressed: what is the size and shape of the PDMS-PEG "vesicle" in the EtOH/H2O mixture. Does it accept more than one Pt complex? DOSY NMR experiments should be carried out to answer this question.

Response:

(a) **(b)**

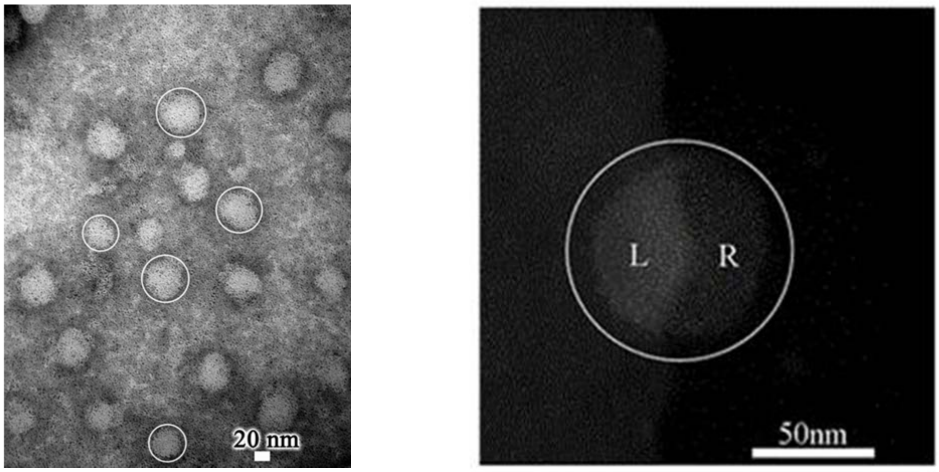

Supplementary Figure 5. TEM (a) and STEM (b) images of Pt_1@PDMS-PEG. For TEM characterization, phosphotungstic acid was used as the staining reagent. From the TEM image, fairly unilamellar vesicles with a narrow distribution (30-80 nm) were obtained. The STEM image of a Pt_1@PDMS-PEG vesicle with the circular area displays a bright region on the left (L) and a less bright region on the right (R). The left side (L) of the vesicle is located at the junction of a copper grid part of the sample holder, and the right side (R) corresponds to the other part on ultrathin carbon film of the sample holder. The average diameter of Pt_1@PDMS-PEG structure in STEM was identical to that observed under TEM. Because the elements in PDMS-PEG are indistinguishable under STEM, and Pt is the only heavy element in the Pt_1@PDMS-PEG solution, the lighted circular areas, both (L) and (R), can be ascribed to Pt_1 atoms encapsulated in the newly formed PDMS-PEG vesicles, and energy dispersive x-ray spectroscopy (EDX) analysis also confirmed the existence of Pt on the vesicle (Supplementary Table 5). Further magnification of the image was not available due to formation of Pt nanoparticles from the aggregation of Pt_1 single atoms, induced by the high energy electron beams (For STEM, no phosphotungstic acid was used).

The TEM and the EDX analysis in STEM have been used to characterize the morphology of Pt_1@PDMS-PEG (**Supplementary Figure 5a**) and the distribution of Pt in the vesicles (**Supplementary Figure 5b** and **Supplementary Table 5**).

The new results and discussions are included in the revised manuscript "The morphology of Pt_1@PDMS-PEG solution was characterized by transmission electron microscopy (TEM). Fairly

unilamellar vesicles with a narrow distribution (30-80 nm, Supplementary Figure 5a) were formed[34]. The energy-dispersive X-ray spectroscopy analysis in a scanning transmission electron microscope (STEM) revealed the existence of Pt in the vesicles (Supplementary Figure 5b and Supplementary Table 5). It should be noted that the Pt atoms tend to agglomerate under intense electron beam or X-ray radiation, making TEM, STEM and synchrotron X-ray measurements infeasible in providing unambiguous evidence of discrete Pt atoms in our system[35]."

The application in hydrosilylation is on the other hand truly outstanding, with much better TOFs and TONs than known systems, which could on its own warrant publication in Nat. Comm. The blank tests need to be carried out. Indeed, if the PDMS has been obtained via hydrosilylation, some Pt might remain in the polymer.

Response: We really appreciate the reviewer's complimentary comment on the performance of hydrosilylation by Pt_1@PDMS-PEG.

The blank test was carried out and reported in the revised manuscript. The blank test procedure was inserted into the "General Procedure for the hydrosilylation of 1-octene catalyzed by Pt_1@PDMS-PEG" in **Methods** as follows "Blank test was run as follows: hydrosilylation reaction was carried out with PDMS-PEG/ethanol-water as the catalyst (without Pt) following the same procedure as for the hydrosilylation of 1-octene catalyzed by Pt_1@PDMS-PEG, No conversion of 1-octene was observed (Supplementary Table 6)."

We also added the results and discussion in the revised manuscript "No 1-octene conversion was observed in a blank test using PDMS-PEG alone (Supplementary Table 6)"

It is claimed that after the catalytic run, the exact same complex is seen by NMR (identical chemical shift). This is rather surprising, as it means that olefin bound Pt complex "(olefin)(R1OR2)PtCl2H2" exists as the resting state in the catalytic process, although it reacts very fast with the silane. Have the authors tried the independent reaction of the complex with silane? What does it lead to?

Response: For Karstedt catalyst, the color of the solution changes from clear to yellow during the reaction, indicating the formation of colloidal platinum species (Advanced Synthesis & Catalysis, 2004, 346, 1429-1434). However, the solution remains colorless and transparent throughout the hydrosilylation reaction with the Pt_1@PDMS-PEG catalyst, indicating high stability of the catalysts during the hydrosilylation and no sign of Pt_1

agglomeration into the colloidal platinum species. The colorless and transparent solution was also noted in the reusability tests.

To further confirm no agglomeration of catalysts, the ^{195}Pt NMR spectrum of the Pt_1@PDMS-PEG after the hydrosilylation reaction [n (1-octene): n(silane) = 5 : 3] was acquired. A possible explanation for why 1-octene still bound to Pt_1 center is added in the caption of **Supplementary Figure 9** "Because of the excess amount of olefin (1-octene), the Pt_1 maintained the (olefin)(R^1OR^2)$PtCl_2H_2$ state (^{195}Pt chemical shift), not $(R^1OR^2)_2PtCl_2H_2$ state." In the revised Manuscirpt, we add the ratio of 1-octene to silane "$n_{1\text{-octene}}$: n_{silane} = 5 : 3"

Lewis *et al.*[44] have reported the aggregation of Karstedt catalyst to form Pt nanoparticles with excessive silane at the end of the hydrosilylation reaction (after full conversion of olefin). In our work, 1-octene was added and mixed with the Pt_1@PDMS-PEG first; see "General Procedure for the hydrosilylation of 1-octene catalyzed by Pt_1@PDMS-PEG" in **Methods**. When silane was added first to a concentrated Pt_1@PDMS-PEG solution, as inquired by the reviewer, the solution turned into a mud-like slurry with light brown color and was not suited for further characterization.

Finally, if the complex is intact after a first catalytic run, reloading and recycling experiments should be performed.

Response: We performance reusability of the catalyst for three times. The results are given in **Supplementary Figure 10**. The discussion is included in the revised manuscript "Moreover, through three recycled uses, Pt_1@PDMS-PEG maintained high activity without noticeable deactivation in 1-octene hydrosilylation with $(Me_3SiO)_2MeSiH$ (Supplementary Figure 10). The color of the reused Pt_1@PDMS-PEG solution remained clear throughout the reuses."

The procedure is given in **Methods**: "After the hydrosilylation reaction between 1-octene and $(Me_3SiO)_2MeSiH$ (1-octene: 0.4488 g, $(Me_3SiO)_2MeSiH$: 0.89 g) finished, the solution was centrifuged. 0.6694 g reaction mixture was taken out from the upper solution, and analyzed by ^1H NMR. The remaining solution (0.6694 g) was used for the subsequent hydrosilylation reaction. For the reuse test, 0.4488 g 1-octene and 0.89 g $(Me_3SiO)_2MeSiH$

was added. After reaction, solution was centrifuged. 1.3388 g (equal to the amount of added reactants) reaction mixture was taken out from the upper solution, and analyzed by ^{1}H NMR, while the remaining solution (0.6694 g) was used for the subsequent hydrosilylation reaction. For the following reuses, the added amounts of 1-octene and $(Me_3SiO)_2MeSiH$ were always 0.4488 g and 0.89 g, respectively. After each reaction, the amount of reaction mixture taken out for ^{1}H NMR from the upper solution after centrifugation was always 1.3388 g (equal to the amount of added reactants), and the remaining solution was kept at 0.6694 g for the subsequent hydrosilylation reaction."

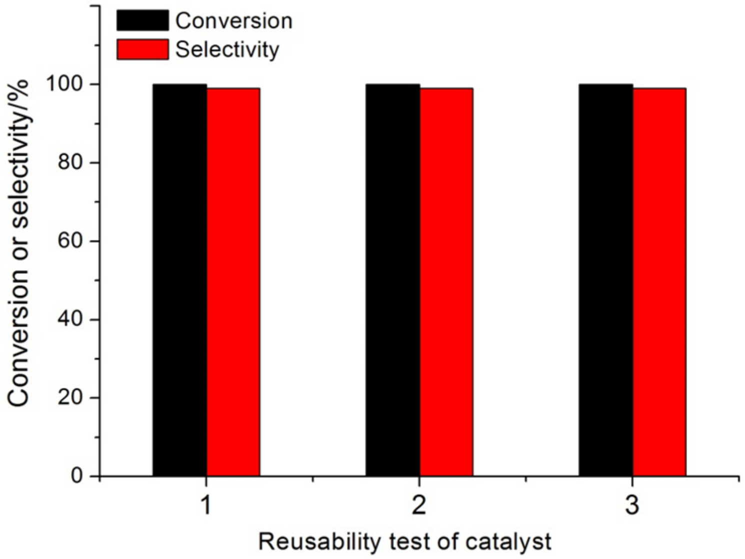

Supplementary Figure 10. Conversion/selectivity vs. number of catalyst reuse of hydrosilylation between 1-octene and $(Me_3SiO)_2MeSiH$ ($n_{1\text{-octene}}$: n_{silane} = 1:1). The reaction conditions were the same as that used for a fresh catalyst (see **Methods**)

In conclusion for the synthesis/characterization of the Pt1 species, the data is not consistent with the proposed structure.

Response: We really appreciated the reviewer's constructive comments and suggestions. Following these suggestions, we have carried out extensively additional experiments and additional calculations focusing on the characterization of Pt_1@PDMS-PEG. Based on new results and analysis, we have reached an upgraded understanding about Pt_1@PDMS-PEG and meanwhile we have rewritten the Introduction and made necessary corrections on some key terminologies used in the previous manuscript that might cause

confusion. We hope the reviewer would find these revisions satisfactory. (The rewritten Introduction in the revised manuscript follows: "Supported single atoms have been prepared using various methods[1,2]. The reduced metal atoms are stabilized on solid surfaces through weak interaction[3,4] or by strong ligand coordination[5,6]. Weakly coordinated noble metal atoms are often capable of activating adsorbed reactants in catalysis[7,8]. Reduced single atoms tend to aggregate into nanoparticles in liquid media in the absence of strong ligand coordination[9]. Full and strong coordination is not desired for metal catalysts[10]. Therefore, synthesis of weakly coordinated and yet stable metal atoms in liquid media may pave a new path for facile preparation of highly dispersed metal catalysts by overcoming the long-standing challenge of metal aggregation. The readily removable ligands from the discrete metal atoms may also potentially enable atomically controllable synthesis of metallic materials by design.

Numerous surfactants with diverse functionalities have been used in the synthesis of metallic nanoparticles[11,12] or metal ion-surfactant complexes[13,14] in micro-emulsions system. The synthesis of discrete reduced metal atoms with weak coordination which are sufficiently stable in storage at moderate temperature has not been reported. In this work, we chose polydimethylsiloxane-polyethylene glycol (PDMS-PEG) copolymer as an amphiphilic solvating agent. The hydrophilic and semicrystalline PEG blocks enable the dispersion of the hydrophobic PDMS blocks in aqueous solutions[15,16]. In our synthesis, ethanol was used as the reductant for Pt(IV)[17,18], in the presence of optimized 10% (v/v) water to accelerate the rate of reduction[9], while the diverse oxygen-containing functionality of PDMS-PEG may offer weak coordination to the Pt atoms. "

In conclusion, I believe that the generation of a highly active Pt complex in PDMS-PEG for hydrosilylation of alkenes is of high interest. However, I am not convinced at all by the structure proposed by the authors, and several additional experiments should be carried out. Comparison with data in the literature for Pt(II) complexes should also be done. Moreover, the introduction needs to be modified in depth.
For these reasons, I recommend rejection of the article, but I believe it would make a nice contribution to Nat. Comm once the points raised are addressed.

Response: Once again, we really appreciated the reviewer's constructive comments and suggestions. Following these suggestions, we have carried out additional experiments

and calculations focusing on the full characterization of Pt_1@PDMS-PEG. We paid extra attention to distinguish Pt(0) and Pt(II) complexes based on the studies of literatures and on the results of our own new experiments and calculation. Our renewed analysis demonstrated the existence of Pt(0) in Pt_1@PDMS-PEG unambiguously. Meanwhile, we have revised the Introduction and made numerous corrections on some key terminologies used the original manuscript that might cause confusion. Together with the original results, newly added results as outlined in the revised manuscript allowed us to acquire a deeper understanding about Pt_1@PDMS-PEG. We hope the reviewers and the editor would find these revisions satisfactory.

Reviewers' comments:

Reviewer #1 (Remarks to the Author):

This paper describes the easy method to prepare the single atom catalyst of Pt. In this method, the use of polydimethylsiloxane-polyethylene glycol (PDMS-PEG) copolymer, which weakly supports the Pt atom, as an amphiphilic solvating agent successfully prevent the aggregation of Pt metals. Formation of the Pt1 species is evidenced by both experimentally and theoretically. When I previously reviewed this article, I expressed some queries. In this submission the manuscript is far improved, and I basically recommend this journal for publication after changing the following points.
1) The electronic state of Pt in the main text is still ambiguous. Especially the use of the word "electron deficient" should be reconsidered. For example, the authors describe "the electron deficient Pt1 in the structure of (R1OR2)2PtCl2H2" in the main text. Then one can imagine the electron deficient Pt(IV) centers in this system. On the other hand, several examinations in this study support the low-valent Pt center not Pt(IV) or Pt(II) of (R1OR2)2PtCl2H2". Do the authors want to describe "electron deficient as the Pt(0)"? This point should be clarify.
2) Related to the above query, the description of (R1OR2)2PtCl2H2 and (R1OR2)2PtCl2(H3O)2 should be reconsidered. As long as the author write the "PtCl2" unit, people systematically imagine the oxidation state of Pt(II). It is recommended that the authors clarify the different bonding nature in their complex systems, let say, using a different description.

Reviewer #2 (Remarks to the Author):

The revised version of the manuscript by Bai, Zhang and co-workers reports on the synthesis of "electron deficient "Pt1" in PDMS-PEG" aggregates.
I am pleased to see that they have modified in depth their introduction in line with my general comments.
The authors have made several very important complementary analyses as suggested.
In particular, they showed that the catalyst can be recycled twice with analogous extremely high efficiency.
The DRIFT spectrum showing the CO stretching band at 2084 cm-1 is particularly relevant to show that a stronger back donation from Pt to CO is present, compared to frequencies reported for Pt(II) complexes (JOMC 2001, 622, 180). This shows reduction to Pt(0).
Experimental data supports two Cl- ligands, a single Pt(0) center, one CO ligand, and elimination of HCl upon base addition, showing the H to be acidic.
The authors favor a pseudo-octahedral geometry with two Pt-H bonds.
A structure for which H bonding occurs between H and H2O (supp info, table 3) has been calculated. Note "d" below table 3 states that the Pt-H bond is longer in this case. The calculated chemical shift is closer to the experimental value than the one for the complex they seem to prefer, that is presented in the article (Figure 2).
They state in the article, page 8, lines 136-141: "In the presence of abundant protons such as in residual H2O and OH groups of the solvent, the protic H in the (R1OR2)2PtCl2H2 could be readily hydrated, e.g. H3O+. The calculated 195Pt chemical shift (-2705 ppm) of (R1OR2)2Pt(0)Cl2(H3O+)2 (Entry 6, Supplementary Table 3) is in an excellent agreement with the experimental shift (-2680 ppm), suggesting that (R1OR2)2Pt(0)Cl2(H3O+)2 may exist as a hydrated form of (R1OR2)2PtCl2H2 in the system."
As they mention themselves, there are several molecules in the medium that are Lewis bases (from PEG, PDMS and solvent), that could therefore well interact with H+ via H bonding. This would also be consistent with the coordination of Cl- H+ to Ir nanoparticles (ref 30).
Furthermore, they point that no Pt-H coupling is seen, which is not consistent with a direct Pt-H interaction.
Have the authors considered structures in which two Cl- are bound to Pt(0), and H+ interacting bound

to Cl- and other Lewis bases (PEG or PDMS, or solvent)?

I believe that this is an important point since the authors propose a pseudo-octahedral geometry for the reduced Pt species.

In conclusion, I think that the present work should be published in Nat. Comm. with minor changes related to the structure of the active species.

Reviewers' comments:

Reviewer #1 (Remarks to the Author):

This paper describes the easy method to prepare the single atom catalyst of Pt. In this method, the use of polydimethylsiloxane-polyethylene glycol (PDMS-PEG) copolymer, which weakly supports the Pt atom, as an amphiphilic solvating agent successfully prevent the aggregation of Pt metals. Formation of the Pt1 species is evidenced by both experimentally and theoretically. When I previously reviewed this article, I expressed some queries. In this submission the manuscript is far improved, and I basically recommend this journal for publication after changing the following points.

1) The electronic state of Pt in the main text is still ambiguous. Especially the use of the word "electron deficient" should be reconsidered. For example, the authors describe "the electron deficient Pt1 in the structure of (R1OR2)2PtCl2H2" in the main text. Then one can imagine the electron deficient Pt(IV) centers in this system. On the other hand, several examinations in this study support the low-valent Pt center not Pt(IV) or Pt(II) of (R1OR2)2PtCl2H2". Do the authors want to describe "electron deficient as the Pt(0)"? This point should be clarify.

Response: We would like thank the reviewer's valuable critics on our original manuscript.

To describe the Pt electronic state clearly, as pointed out by reviewer 1, we have changed the expression from "electron deficient Pt_1" to "electron deficient $Pt_1(0)$" (where appropriate, we use $Pt_1^{\delta+}$ ($0<\delta^+<1$)) in the manuscript and supplementary information. We have shown via UV-Vis, FTIR, ^{195}Pt and ^{13}C NMR and XPS spectroscopies that the discrete and reduced state of $Pt_1(0)$ featuring characteristics of electron deficiency $Pt_1^{\delta+}$ ($0<\delta^+<1$).

2) Related to the above query, the description of (R1OR2)2PtCl2H2 and (R1OR2)2PtCl2(H3O)2 should be reconsidered. As long as the author write the "PtCl2" unit, people systematically imagine the oxidation state of Pt(II). It is recommended that the authors clarify the different bonding nature in their complex systems, let say, using a different description.

Response:

Following the reviewer's suggestion, we have changed the description $(R^1OR^2)_2PtCl_2H_2$ and $(R^1OR^2)_2PtCl_2(H_3O)_2$ to $(R^1OR^2)_2Pt(0)Cl^-{}_2H^+{}_2$ and $(R^1OR^2)_2Pt(0)Cl^-{}_2(H_3O^+)_2$ to show that the Pt_1 center is in a reduced state, while Cl and H are ions.

Reviewer #2 (Remarks to the Author):

The revised version of the manuscript by Bai, Zhang and co-workers reports on the synthesis of "electron deficient "Pt1" in PDMS-PEG" aggregates.
I am pleased to see that they have modified in depth their introduction in line with my general comments.
The authors have made several very important complementary analyses as suggested.
In particular, they showed that the catalyst can be recycled twice with analogous extremely high efficiency.
The DRIFT spectrum showing the CO stretching band at 2084 cm-1 is particularly relevant to show that a stronger back donation from Pt to CO is present, compared to frequencies reported for Pt(II) complexes (JOMC 2001, 622, 180). This shows reduction to Pt(0).
Experimental data supports two Cl- ligands, a single Pt(0) center, one CO ligand, and elimination of HCl upon base addition, showing the H to be acidic.

The authors favor a pseudo-octahedral geometry with two Pt-H bonds.
A structure for which H bonding occurs between H and H2O (supp info, table 3) has been calculated. Note "d" below table 3 states that the Pt-H bond is longer in this case. The calculated chemical shift is closer to the experimental value than the one for the complex they seem to prefer, that is presented in the article (Figure 2).

They state in the article, page 8, lines 136-141: "In the presence of abundant protons such as in residual H2O and OH groups of the solvent, the protic H in the (R1OR2)2PtCl2H2 could be readily hydrated, e.g. H3O+. The calculated 195Pt chemical shift (-2705 ppm) of (R1OR2)2Pt(0)Cl2(H3O+)2 (Entry 6, Supplementary Table 3) is in an excellent agreement with the experimental shift (-2680 ppm), suggesting that (R1OR2)2Pt(0)Cl2(H3O+)2 may exist as a hydrated form of (R1OR2)2PtCl2H2 in the system."
As they mention themselves, there are several molecules in the medium that are Lewis bases (from PEG, PDMS and solvent), that could therefore well interact with H+ via H bonding. This would also be consistent with the coordination of Cl- H+ to Ir nanoparticles (ref 30).

Furthermore, they point that no Pt-H coupling is seen, which is not consistent with a direct Pt-H interaction.

Response:

The understanding of reviewer 2 is correct. Indeed, Pt-H coupling was not observed, likely due to the fast exchanges between anhyrdated and hydrated form of $(R^1OR^2)_2PtCl_2H_2$.

Have the authors considered structures in which two Cl- are bound to Pt(0), and H+ interacting bound to Cl- and other Lewis bases (PEG or PDMS, or solvent)?

Response:

We have further calculated ^{195}Pt NMR parameters for the structures ($Pt(HCl)_2$ and $Pt(HCl)_2(C_2H_5OH)_2$) as suggested by the reviewer and the results are given in Supplementary

Table 3 (Entries 14 and 15). As expected, the calculated ^{195}Pt chemical shifts do not agree with the experimental value (-2680 ppm). In the revised Supplementary Materials, we added statement of "We also considered the structures in which two Cl^- are bound directly to Pt(0) while H^+ is bound to Cl^- and between Cl^- and the hydroxyl oxygen of ethanol (Lewis bases) (Entry 14 and 15, Supplementary Table 3). Both structures failed to produce the matches to the observed ^{195}Pt chemical shift. It is noted that the structure ($Pt(HCl)_2$) (Entry 14, ΔE = -2.4 eV) is much less stable than the $Pt(0)Cl^-_2H^+_2$ (Entry 1, ΔE = -4.0 eV)."

In the revised manuscript, we emphasized this result by adding a phase in the sentence on page 7 as: "We noted that various oxygen coordinated structures, excluding Cl and H and the structures in which H^+ is bound to Cl^- failed to produce a structure matching the observed ^{195}Pt chemical shift (Supplementary Table 3)."

I believe that this is an important point since the authors propose a pseudo-octahedral geometry for the reduced Pt species.

In conclusion, I think that the present work should be published in Nat. Comm. with minor changes related to the structure of the active species.
